# Insight into the metabolic mechanism of Diterpene Ginkgolides on antidepressant effects for attenuating behavioural deficits compared with venlafaxine

**DOI:** 10.1038/s41598-017-10391-1

**Published:** 2017-08-29

**Authors:** Shunjie Bai, Xiaodong Zhang, Zhi Chen, Wei Wang, Qingchuan Hu, Zihong Liang, Peng Shen, Siwen Gui, Li Zeng, Zhao Liu, Jianjun Chen, Xiongfei Xie, Hua Huang, Yu Han, Haiyang Wang, Peng Xie

**Affiliations:** 1 Department of Neurology, Yongchuan Hospital, Chongqing Medical University, Chongqing, China; 2 Chongqing Key Laboratory of Neurobiology, Chongqing, China; 3 Institute of Neuroscience and the Collaborative Innovation Center for Brain Science, Chongqing Medical University, Chongqing, China; 4 Key Laboratory of Laboratory Medical Diagnostics of Education, Department of Laboratory Medicine, Chongqing Medical University, Chongqing, China; 5 Department of Neurology, The Inner Mongolia Autonomous Region people’s Hospital, Hohhot, Inner Mongolia, China; 6 Department of Neurology, The First Affiliated Hospital of Chongqing Medical University, Chongqing, China; 7 Department of Radiology, The First Affiliated Hospital of Chongqing Medical University, Chongqing, China; 8 Department of Neurology, The Second Affiliated Hospital of Chongqing Medical University, Chongqing, China

## Abstract

Depression is a severe and chronic mental disorder, affecting about 322 million individuals worldwide. A recent study showed that diterpene ginkgolides (DG) have antidepressant-like effects on baseline behaviours in mice. Here, we examined the effects of DG and venlafaxine (VLX) in a chronic social defeat stress model of depression. Both DG and VLX attenuated stress-induced social deficits, despair behaviour and exploratory behaviour. To elucidate the metabolic changes underlying the antidepressive effects of DG and VLX, we investigated candidate functional pathways in the prefrontal cortex using a GC-MS-based metabolomics approach. Metabolic functions and pathways analysis revealed that DG and VLX affect protein biosynthesis and nucleotide metabolism to enhance cell proliferation, with DG having a weaker impact than VLX. Glutamate and aspartate metabolism played important roles in the antidepressant effects of DG and VLX. Tyrosine degradation and cell-to-cell signaling and interaction helped discriminate the two antidepressants. L-glutamic acid was negatively correlated, while hypoxanthine was positively correlated, with the social interaction ratio. Understanding the metabolic changes produced by DG and VLX should provide insight into the mechanisms of action of these drugs and aid in the development of novel therapies for depression.

## Introduction

Depression is a severe and chronic mental disorder, affecting about 322 million people of all ages worldwide, equivalent to 4.4% of the world’s population^1–4^. Although numerous antidepressants have been approved to treat depressive symptoms, their efficacy is limited and they have various side effects, including an increased risk of suicide in patients treated with some of these medications^5, 6^. Owing to these limitations of conventional antidepressants, treatment of depressive disorders remains a major focus of current medical research, and studies of alternative treatments are urgently required.

Natural medicinal products are now recognized as a major source of effective modern medicines^7^. Natural medicines have various therapeutic effects, including the ability to detoxify, as well as anti-inflammatory and anti-oxidative actions^8, 9^. Accumulating evidences indicates that diterpene ginkgolides (DG; mainly consisting of ginkgolides A, B and K) are primarily responsible for the neuroprotective actions of *Ginkgo biloba* extract^10–14^. Indeed, a recent study by our group showed that DG has antidepressant-like effects on baseline behaviours in mice^14^. Major depressive disorder is characterized by a chronically dysregulated stress response, and stress has been shown to be a major factor contributing to the development of depression^15^. Therefore, it is necessary to evaluate the effect of DG in a rodent model of depression. The antidepressive effects of venlafaxine (dual reuptake inhibitor of serotonin and norepinephrine) has been shown previously in rodents by our group as well as investigators at other research institutes^16–18^. Additionally, venlafaxine has been found to exhibit antidepressant activities on typical depression-like behaviours, social avoidance, and anxiety-like behaviours induced by chronic social defeat stress (CSDS) after repeated administration^17^. However, the functional and molecular bases of venlafaxine’s antidepressive action in the CSDS model have not been fully clarified. Unbiased overall metabolic profiling may shed new light on the molecular mechanisms targeted by both established and experimental pharmacotherapies^19–22^, thereby facilitating the development of novel antidepressant treatments that target defined metabolic pathways.

Identifying the brain regions that are functionally affected by antidepressants is necessary for understanding the mechanisms of action of these drugs^23^. The prefrontal cortex (PFC) is a critical neuroanatomical hub that controls motivated behaviours in mammals^24, 25^. Structural, functional and metabolic changes in the PFC are key features of rodent models of depression, and accordingly, it has been the focus of studies on the molecular pathogenesis of the disease^21, 26, 27^. Furthermore, the PFC is not only altered in depressed patients and stressed animals, but is also affected by antidepressants^23, 28, 29^.

Here, we compared the effects of DG and VLX in mice subjected to CSDS, an ethologically validated model of chronic stress and depression^30–32^. We examined whether administration of DG and VLX could modulate stress-induced behavioural deficits. To elucidate the metabolite changes that may underlie the pharmacological effects of DG and VLX, we investigated candidate functional pathways in the PFC using a metabolomics approach based on GC-MS technology. Investigating the role of DG and VLX in the PFC may offer important novel mechanistic insight into their therapeutic actions and facilitate the development of novel antidepressant drugs.

## Results

### CSDS induced behavioural deficits

**Figure 1:**
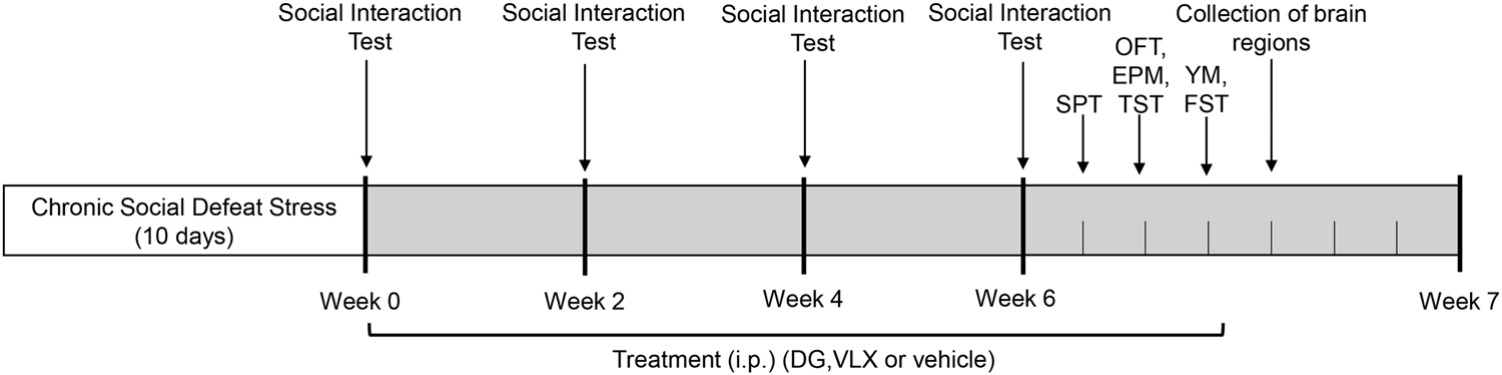
Schedule of chronic social defeat stress (10 days), social interaction test, drug treatment, and behavioural tests. i.p., injection intraperitoneal; DG, diterpene ginkgolides; VLX, venlafaxine; SPT, sucrose preference test; OFT, open field test; EPM, elevated plus maze; TST, tail suspension test; YM, Y-maze; FST, forced swim test.

**Figure 2:**
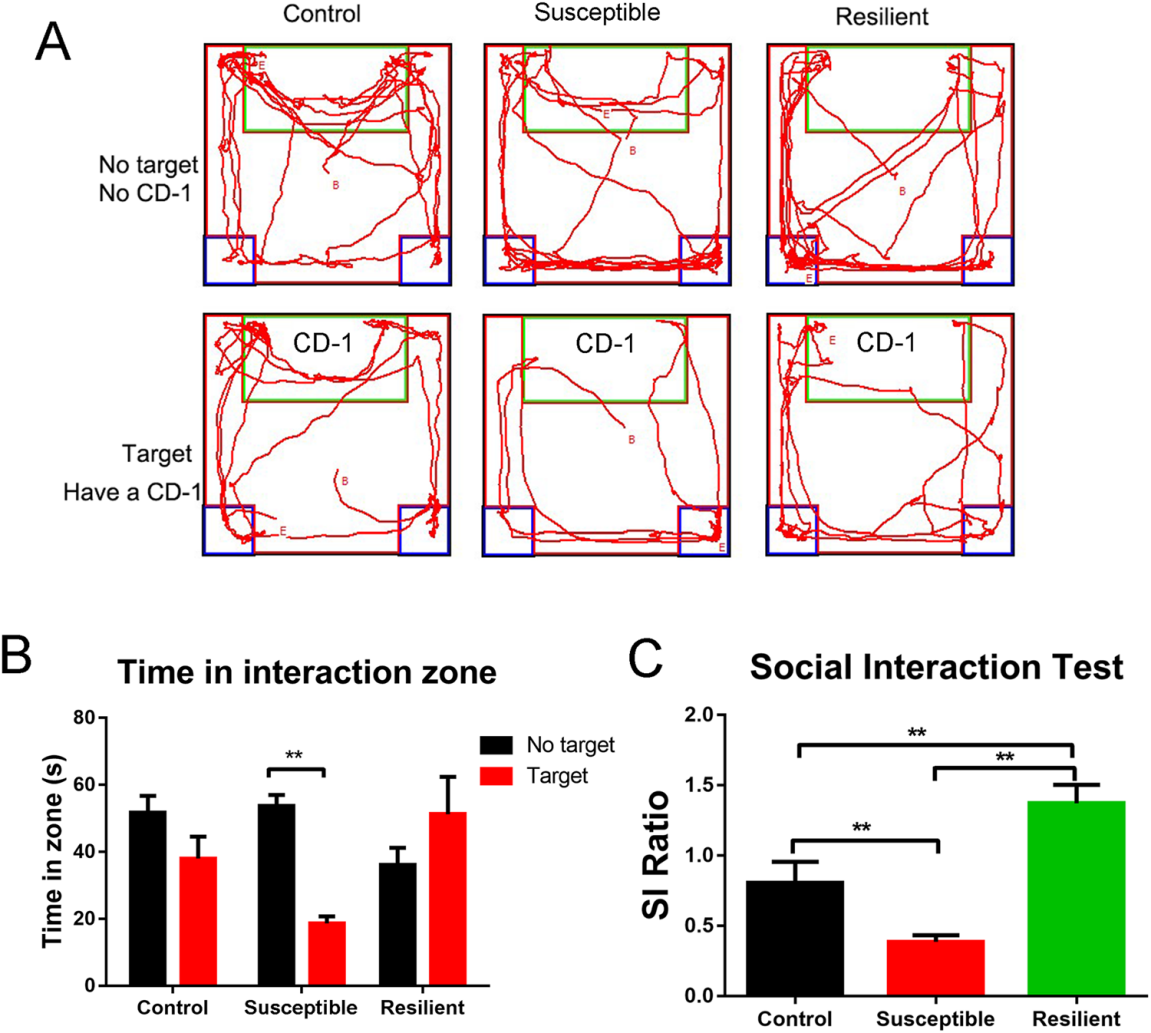
The results of the SI test after the 10-day chronic social defeat stress procedure. (**A**) Mouse track examples in the SI behaviour test. (**B**) Time spent in the interaction zone for control (*n* = 10), susceptible (*n* = 31), and resilient (*n* = 6) mice. The susceptible mice spent less time in the interaction zone when CD-1 mouse was present than when CD1 was absent. **P = 0.000 (Target vs. No target) for susceptible mice, by two-tailed t-test. (**C**) The control (*n* = 10), susceptible (*n* = 31) and resilient (*n* = 6) mice were divided according to their SI ratio. **P = 0.001 (susceptible vs. control); **P = 0.002 (resilient vs. control); **P = 0.000 (resilient vs. susceptible), by one-way ANOVA, followed by post hoc least significant difference test. All data are represented as mean ± SEM, *P < 0.05 and **P < 0.01.

Schedule of the experimental approach is shown in Fig. 1. In accordance with previous studies^32, 33^, mice subjected to 10-day CSDS exhibited sociability deficits. The social interaction (SI) behavioural test was used to evaluate the susceptible (SI ratio < 1) and resilient (SI ratio > 1) phenotypes of defeated mice. As the track (Fig. 2A) and the time in the interaction zone (Fig. 2B) indicated, the susceptible mice spent less time in the interaction zone when a CD-1 mouse, “a novel aggressor mouse”, was present than when the CD1 mouse was absent. The SI ratio was lower in susceptible mice than in the control and resilient groups (Fig. 2C). 31 (77.5%) out of a total of 40 defeated mice showed susceptible or depressed behavior, and 6 mice (15.0%) showed resilient behavior, according to their SI ratio. 3 mice (7.5%) were excluded from the study because of physical wounding. Only 31 susceptible mice among the 40 defeated mice were used for all further experiments. The susceptible mice were randomly divided into vehicle-treated group (DEP, *n = *10), DG-treated group (*n = *11) and VLX-treated group (*n = *10).

### DG and VLX reverse specific stress-induced behavioural deficits

**Figure 3:**
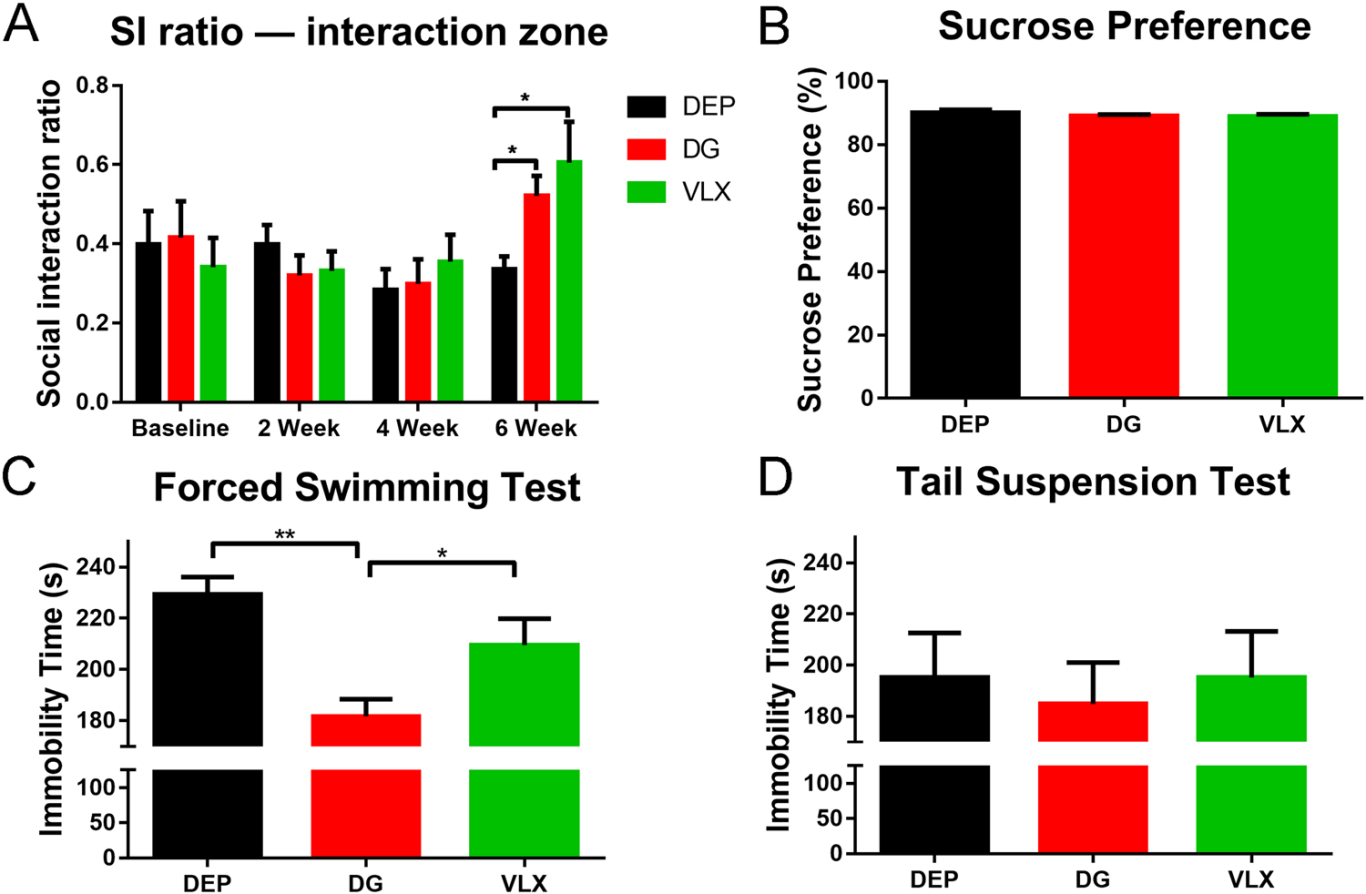
Effects of DG and VLX on behavioural changes in depressed mice after treatment. (**A**) SI ratio at baseline and after 2, 4, and 6 weeks of treatment. Until week 6, VLX (*n* = 10) and DG-treated (*n* = 11) depressed mice exhibited a higher SI ratio compared with DEP (*n* = 10) mice. *P = 0.021 (DG vs. DEP); *P = 0.010 (VLX vs. DEP), by one-way ANOVA, followed by post hoc Dunnett’s test. (**B**) Sucrose preference after 6 weeks of treatment (*n:* DEP = 10, DG = 11, VLX = 10). (**C**) The immobility time in the FST after 6 weeks of treatment. DG-treated (*n* = 11) mice exhibited less immobility time compared with DEP (*n* = 10) mice or VLX (*n* = 10) mice. **P = 0.000 (DG vs. DEP); *P = 0.025 (DG vs. VLX), by one-way ANOVA, followed by post hoc least significant difference test. (**D**) Immobility time in the TST after 6 weeks of treatment (*n:* DEP = 10, DG = 11, VLX = 10). All data are represented as mean ± SEM, *P < 0.05 and **P < 0.01. DEP, vehicle-treated mice; DG, diterpene ginkgolides-treated mice; VLX, venlafaxine-treated mice.

In our previous study, we found that two weeks of treatment with DG produced antidepressant-like effects^14^. In the present study, the SI test was carried out after treatment for 2, 4 and 6 weeks. Until week 6, VLX and DG-treated depressed mice exhibited a higher SI ratio compared with vehicle-treated (DEP) mice (Fig. 3A), indicating an improvement of stress-induced social deficits. However, the DEP group still exhibited pronounced avoidance of the interaction zone. The sucrose preference test (SPT) is a reward-based test, used as an indicator of anhedonia. We previously demonstrated that susceptible mice subjected to CSDS exhibit decreased sucrose preference^33^. This reduced preference for sucrose was not abrogated by treatment with antidepressants (Fig. 3B). The immobility time of the DG group was significantly less than that of DEP group in the FST (Fig. 3C). However, only a tendency for a reduction of the immobility time was shown in the tail suspension test (TST) (Fig. 3D), indicating a partial antidepressant effect of DG on despair behavior. These results suggest that chronic DG and VLX treatments have antidepressant effects in the CSDS model.

### Effects of DG and VLX on exploration, locomotion, anxiety and memory behaviours

**Figure 4:**
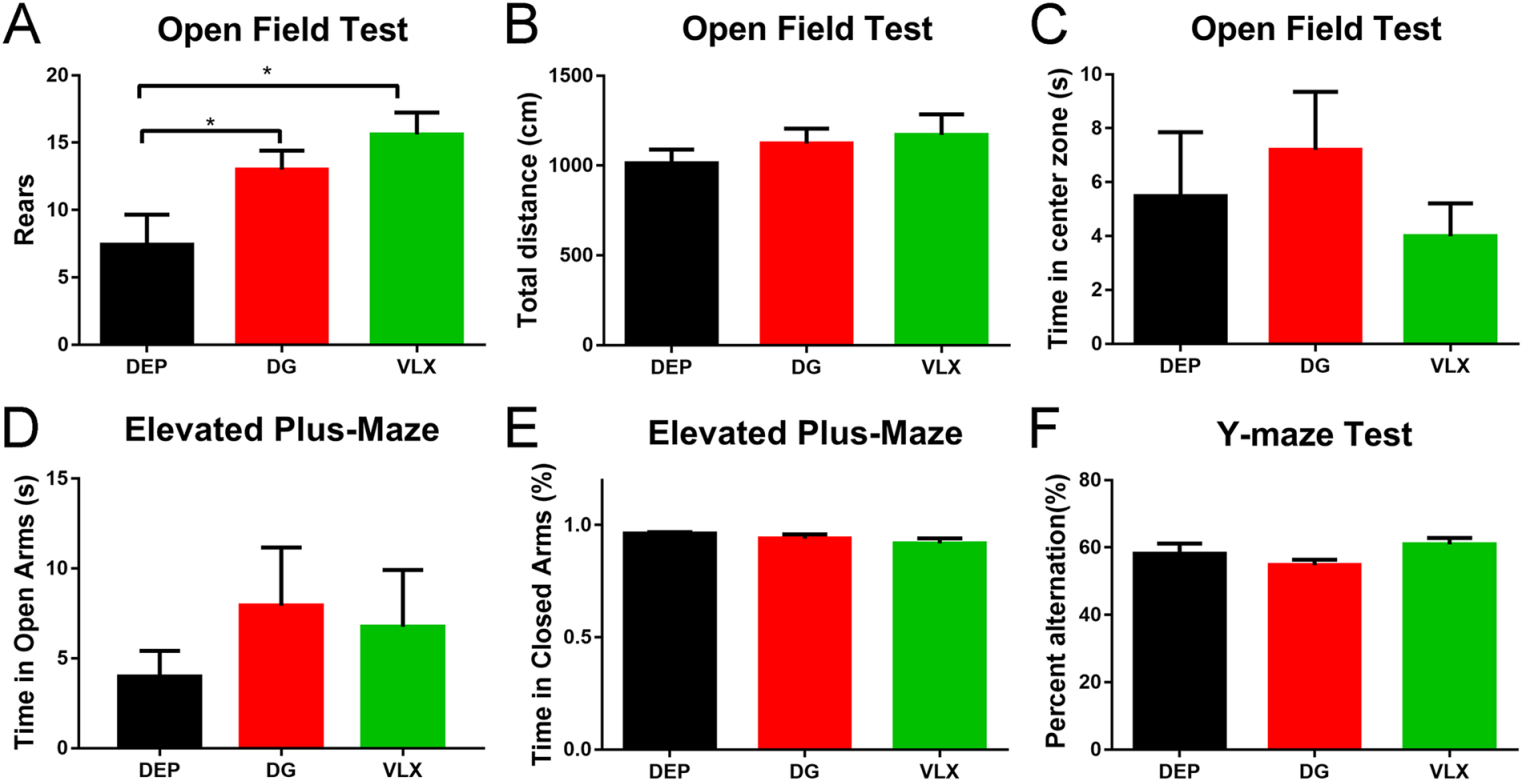
Behavioural results after 6 weeks of treatment. **(A)** Rearings in the OFT after 6 weeks of treatment. DG and VLX increased the number of rearings in depressed mice (*n:* DEP = 10, DG = 11, VLX = 10). *P = 0.036 (DG vs. DEP); *P = 0.029 (VLX vs. DEP), by one-way ANOVA, followed by post hoc least significant difference test. (**B**) Locomotion in the open field (*n:* DEP = 10, DG = 11, VLX = 10). (**C**) The time spent in the centre of the open field (*n:* DEP = 10, DG = 11, VLX = 10). **(D)** The time spent in the open arm in the EPM (*n:* DEP = 10, DG = 11, VLX = 10). (**E**) The percentage of time spent in the closed arm in the EPM (*n:* DEP = 10, DG = 11, VLX = 10). (**F**) The percentage of alternations in the Y-maze (*n:* DEP = 10, DG = 11, VLX = 10). All data are represented as mean ± SEM, *P < 0.05. DEP, vehicle-treated mice; DG, diterpene ginkgolides-treated mice; VLX, venlafaxine-treated mice.

CSDS also induckes anxiety-like behaviour and deficits in exploration^33, 34^. In the open field test (OFT), DG and VLX increased rearing behaviour (Fig. 4A), indicating an amelioration of the exploration deficits. Overall, there was no main effect of treatment on locomotion or time spent in the centre of the open field (Fig. 4B,C). This suggests that DG and VLX have no effect on locomotor activity or anxiety-like behaviour. The elevated plus maze (EPM) is another widely used anxiety paradigm. However, there was no significant difference in this test either, indicating no anxiolytic-like effect of DG or VLX administration (Fig. 4D,E). The Y-maze is a hippocampus-dependent spatial working memory task. There was no significant difference in the percentage of spontaneous alternations (Fig. 4F), indicating DG and VLX might have no influence on spatial memory.

### OPLS-DA model of GC-MS metabolomic analysis

**Figure 5:**
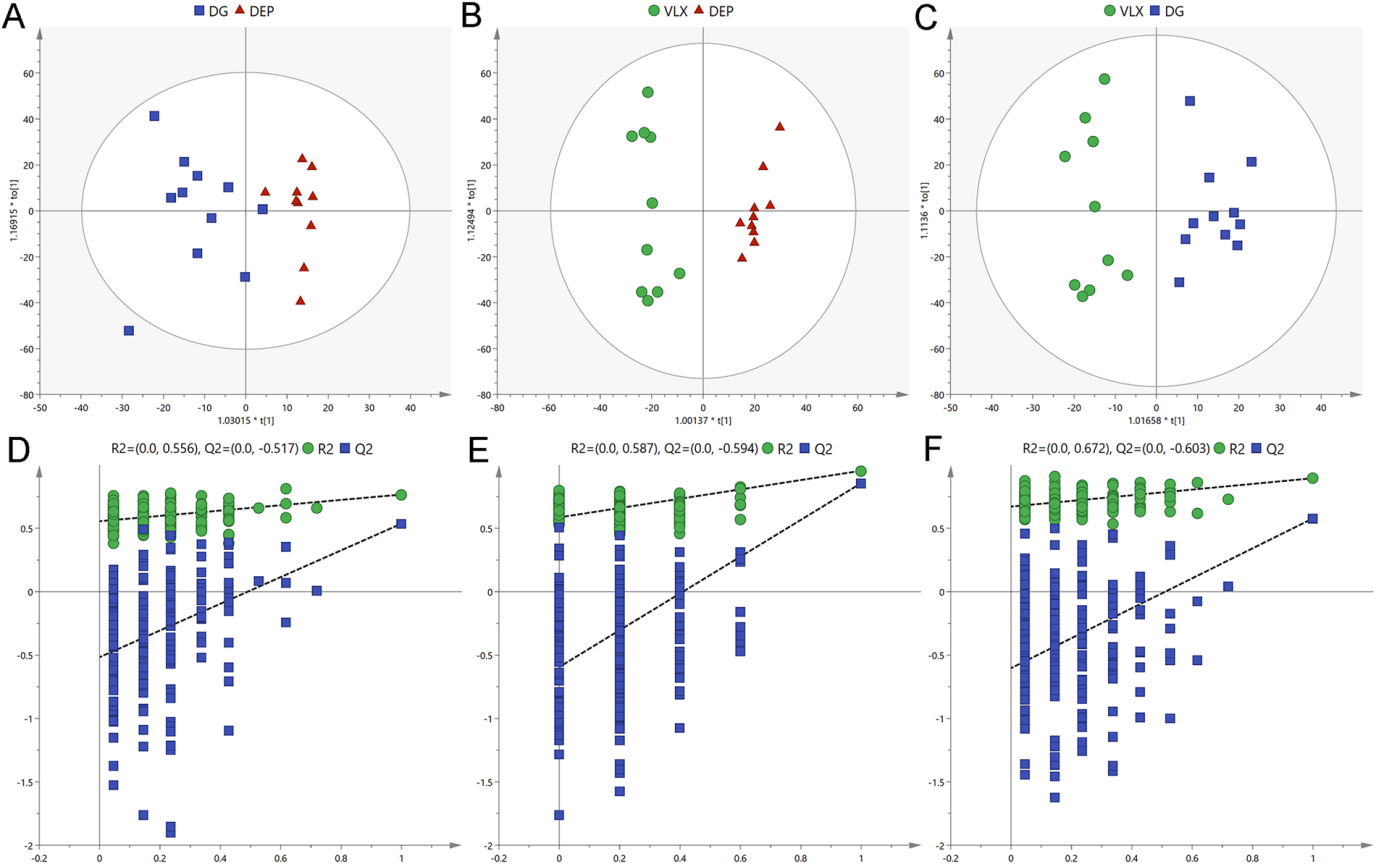
Metabolomic analysis of PFC samples from DG-treated (*n* = 11), VLX-treated (*n* = 10), and saline-treated (DEP; *n* = 10) mice. OPLS-DA score plots for pairwise comparisons between DG and DEP (**A**), VLX and DEP (**B**), and DG and DEP (**C**). Statistical validation of the OPLS-DA model by permutation testing between DG and DEP (**D**), VLX and DEP (**E**), and DG and DEP (**F**). DEP, vehicle-treated mice; DG, diterpene ginkgolides-treated mice; VLX, venlafaxine-treated mice.

In the PCA score plots (Supplemental Figure S2), the R^2^X values were 0.73, indicating the presence of distinct metabolic profile patterns that can be attributed to the specific effects of DG and VLX treatment. These differences could be attributed to changes in levels of metabolites. Then we used the metabolites from the pairwise groups (11 DG mice vs. 10 DEP mice; 10 VLX mice vs. 10 DEP mice, and 11 DG mice vs. 10 DEP mice) to build OPLS-DA models. The pairwise OPLS–DA score plots demonstrated clear discriminations between the DG and DEP groups (R^2^X = 0.712, R^2^Y = 0.955, Q^2^ = 0.857; Fig. 5A), the VLX and DEP groups (R^2^X = 0.47, R^2^Y = 0.767, Q^2^ = 0.537; Fig. 5B), and the DG and VLX groups (R^2^X = 0.649, R^2^Y = 0.895, Q^2^ = 0.579; Fig. 5C). Furthermore, the results of 200-iteration permutation test showed that there were no overfitting, and the models were valid and not overfit (Fig. 5D–F).

### Differential metabolites

**Figure 6:**
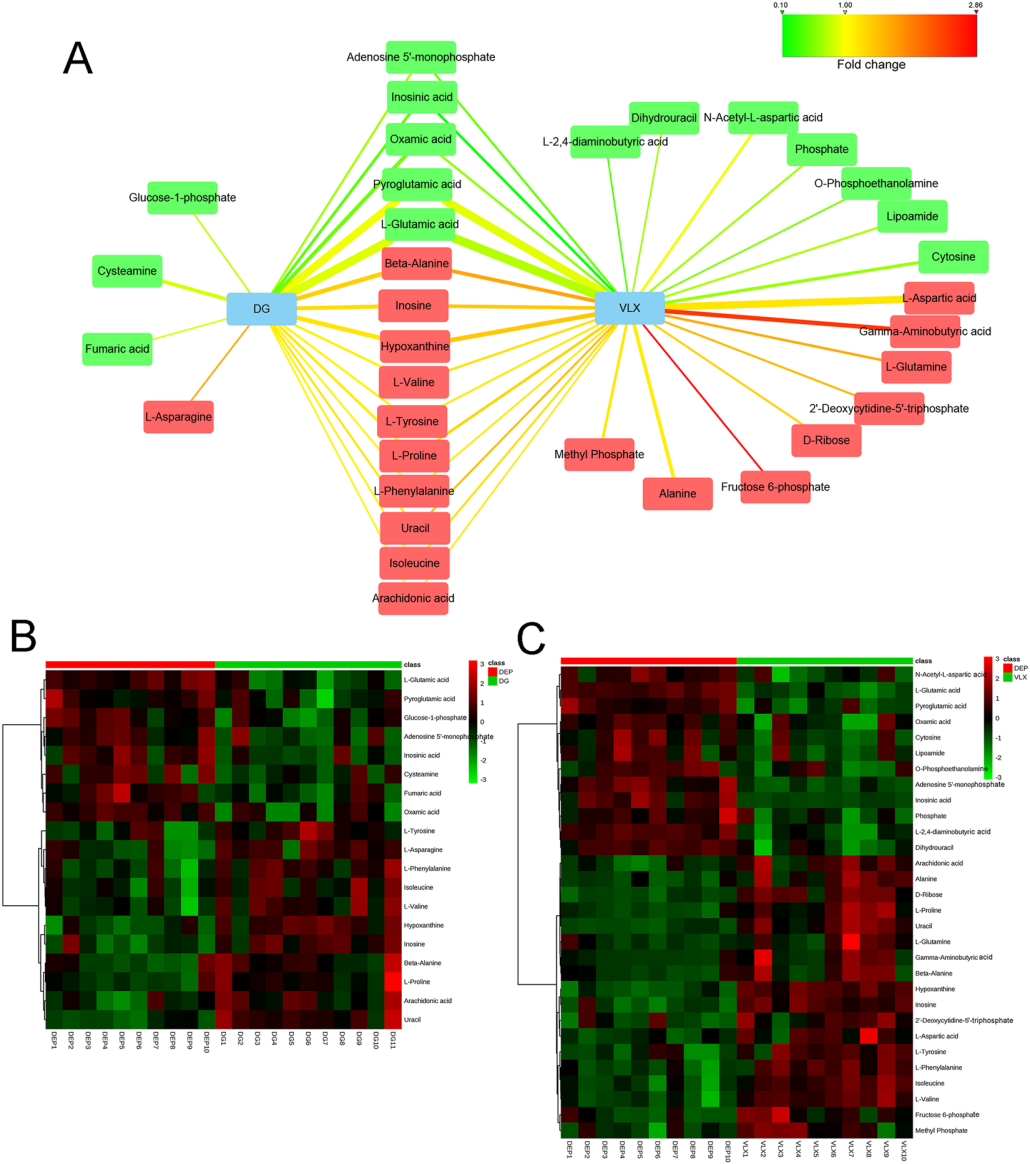
(**A**) Correlation network between the differential metabolites and DG or VLX. Red-coloured boxes indicate upregulation in the DG or VLX group compared with DEP, while green-coloured boxes indicate downregulation in the DG or VLX group, compared with DEP. The line colours represent the fold changes of differential metabolites. The line width represents the VIP scores of the differential metabolites. (**B**) Heat map the differential metabolites in the comparison of the DG and DEP groups. (**C**) Heat map of differential metabolites in the comparison of the VLX and DEP groups. DG, diterpene ginkgolides; VLX, venlafaxine.

A total of 19 differential metabolites (VIP > 1.0 and *P* < 0.05) were identified between the DG and DEP groups (Fig. 6A,B; Supplemental Table S1). Compared with the DEP group, 11 metabolites were upregulated in the DG group, and 8 metabolites were downregulated in the DG group compared with the DEP group. 30 differential metabolites (VIP > 1.0 and *P* < 0.05) responsible for distinguishing VLX-treated mice from the vehicle treated mice were identified (Fig. 6A,C; Supplemental Table S2). Compared with the DEP group, 18 metabolites were upregulated in the VLX group, and 12 metabolites were downregulated in the VLX group relative to the DEP group. Relative to the DEP group, the changes of 15 metabolites in the DG group were same as those in the VLX group; 4 metabolites were specific for DG, attributable to the antidepressant effect, and 15 metabolites were specific for VLX. These results indicate that DG and VLX might exert antidepressive effects through both common and unique molecular pathways. We also compared the differential metabolites between the DG group and the VLX group. Compared with the VLX group, 9 metabolites were upregulated and 8 metabolites were downregulated in the DG group (Supplemental Table S3).

### Ingenuity pathways analysis (IPA) of differential metabolites

**Figure 7:**
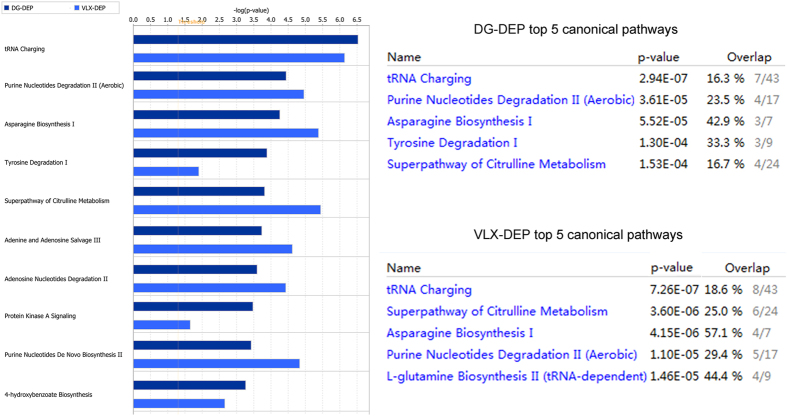
Top canonical pathways enriched in the PFC for comparisons of the DG vs. DEP and the VLX vs. DEP groups. DEP, vehicle-treated mice; DG, diterpene ginkgolides-treated mice; VLX, venlafaxine-treated mice.

**Figure 8:**
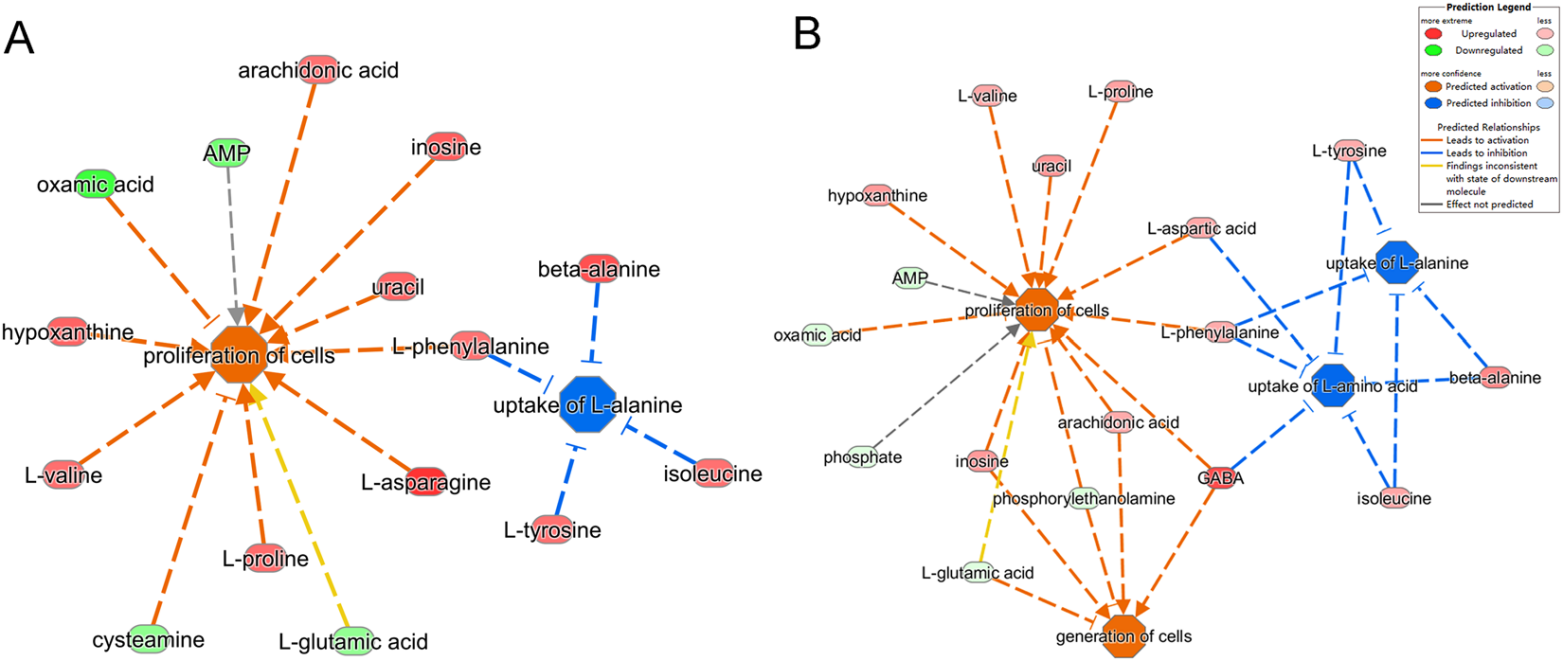
Predicted biological functions (z-score > 2 or z-score <−2) associated with DG (**A**) or VLX (**B**) treatment identified by ingenuity pathways analysis.

To understand the molecular pathways and biological functional roles of the differential metabolites, they were mapped into the IPA knowledgebase. The differential metabolites in DG-treated mice were involved in the following top 5 canonical pathways: tRNA charging, purine nucleotides degradation II (aerobic), asparagine biosynthesis I, tyrosine degradation I, and superpathway of citrulline metabolism. The differential metabolites in the VLX-treated mice were involved in the following top 5 canonical pathways: tRNA charging, superpathway of citrulline metabolism, asparagine biosynthesis I, purine nucleotides degradation II (aerobic), and l-glutamine biosynthesis II (tRNA-dependent) (Fig. 7). The predicted biological functions of the DG-related differential metabolites were significantly involved in the “proliferation of cells” (z-score = 2.528) and the inhibition of the “uptake of l-alanine” (z-score = −2.000) (Fig. 8A). The VLX-related differential metabolites were involved in these two predicted biological functions as well (z-score = 2.867 and −2.000, respectively). In addition, VLX was also involved in the “generation of cells” (z-score = 2.213) and in the inhibition of the “uptake of l-amino acid” (z-score = −2.433) (Fig. 8B). Furthermore, DG’s functional impact in the “growth of organism” (z-score = −2.639) was weaker than VLX (Supplemental Figure S4).

IPA network analysis uses the proprietary ingenuity knowledgebase, and constructs networks of altered molecules which are not limited by canonical pathway boundaries. One DG-related network was constructed (shown in Supplementary Figure S5): “cellular compromise, lipid metabolism, small molecule biochemistry”. Two VLX-related networks were constructed (shown in Supplementary Figures S6 and S7): “amino acid metabolism, small molecule biochemistry, cellular compromise” and “energy production, molecular transport, nucleic acid metabolism”.

### Enrichment analysis and pathway analysis on MetaboAnalyst

**Figure 9:**
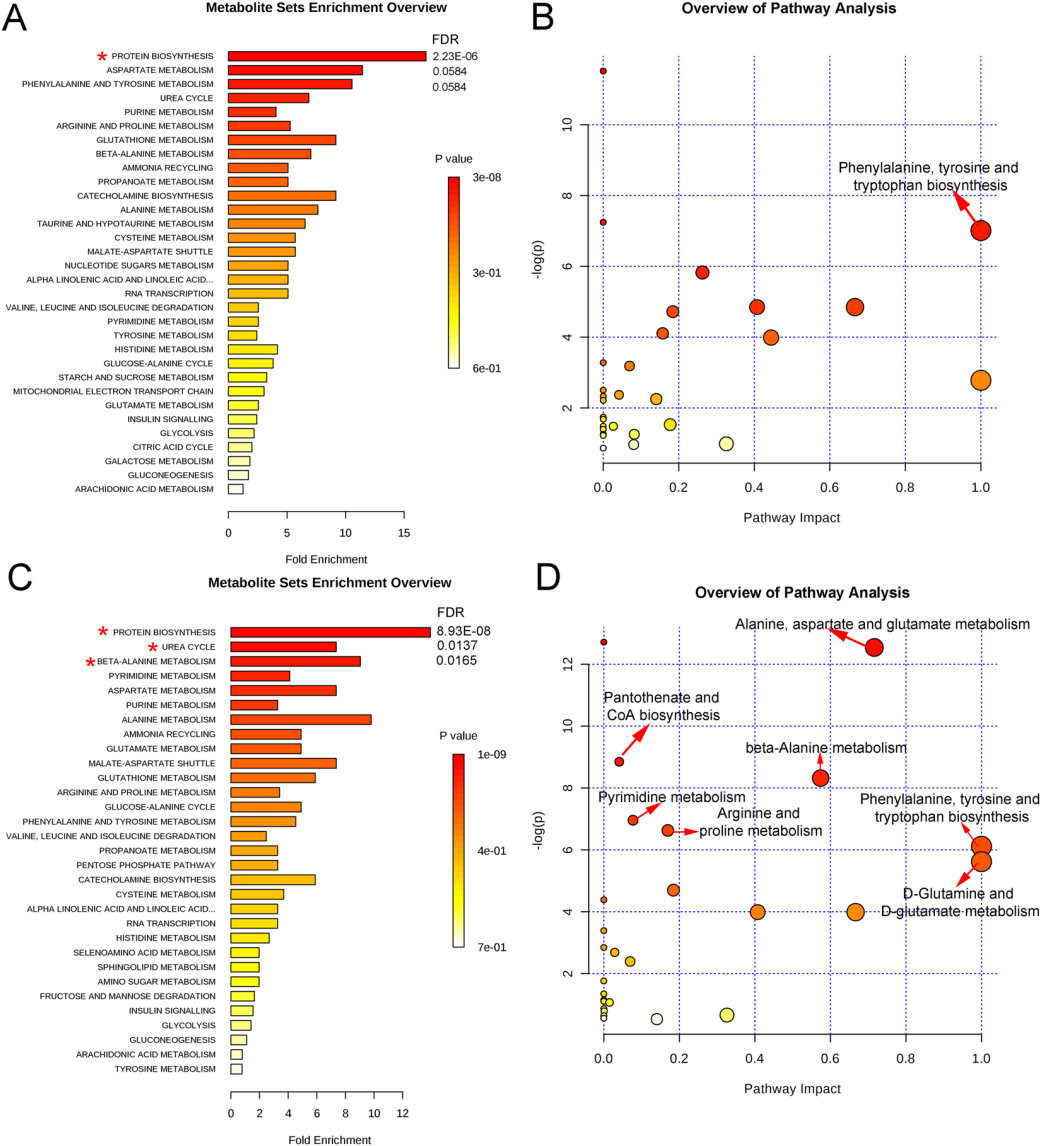
Significant biological functions (**A**) and pathways (**B**) of differential metabolites associated with DG treatment. Significant biological functions (**C**) and pathways **(D)** of differential metabolites associated with VLX treatment. *False discovery rate (FDR) < 0.05; ^→^FDR < 0.05 and impact value > 0.

**Figure 10:**
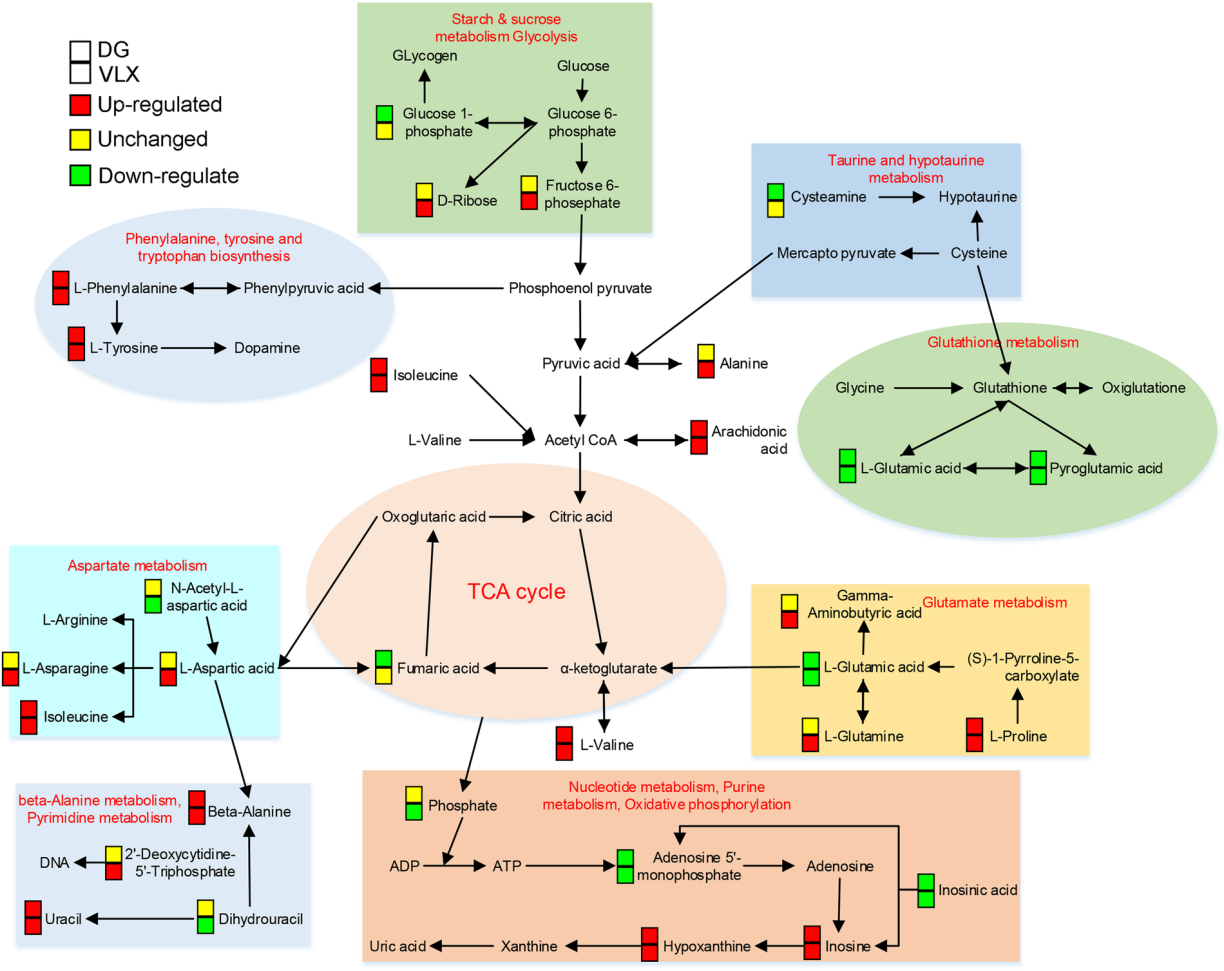
A simplified schematic diagram of the metabolic changes induced by DG and VLX treatment. Red-coloured boxes indicate upregulation, yellow-coloured boxes indicate no significant change, while green-coloured boxes indicate downregulation in the DG or VLX group when compared with DEP group respectively. DG, diterpene ginkgolides; VLX, venlafaxine. TCA, tricarboxylic acid; ADP, adenosine diphosphate; ATP, adenosine triphosphate.

MetaboAnalyst was used to identify altered metabolic pathways and functional roles based on the KEGG metabolic library. According to the following criteria: false discovery rate (FDR) < 0.05 and impact value > 0, the metabolomic alterations induced by DG were enriched in the function of “protein biosynthesis” (Fig. 9A), and the pathway of “phenylalanine, tyrosine and tryptophan biosynthesis” (Fig. 9B). In comparison, the metabolomic changes induced by VLX were enriched in the function of “protein biosynthesis, urea cycle and beta-alanine metabolism” (Fig. 9C), and the pathway of “phenylalanine, tyrosine and tryptophan biosynthesis, D-glutamine and D-glutamate metabolism, alanine, aspartate and glutamate metabolism, beta-alanine metabolism, pantothenate and CoA biosynthesis, pyrimidine metabolism and arginine and proline metabolism” (Fig. 9D). Additionally, compared with VLX, the metabolomic alterations produced by DG were enriched in the function of “protein biosynthesis”, and the pathway of “alanine, aspartate and glutamate metabolism” (Supplementary Figure S7). Based on the functional roles and pathways of all differential metabolites identified by this analysis, a schematic model depicting the potentially altered metabolic pathways was prepared (Fig. 10).

### Correlations between the SI ratio and metabolites

**Figure 11:**
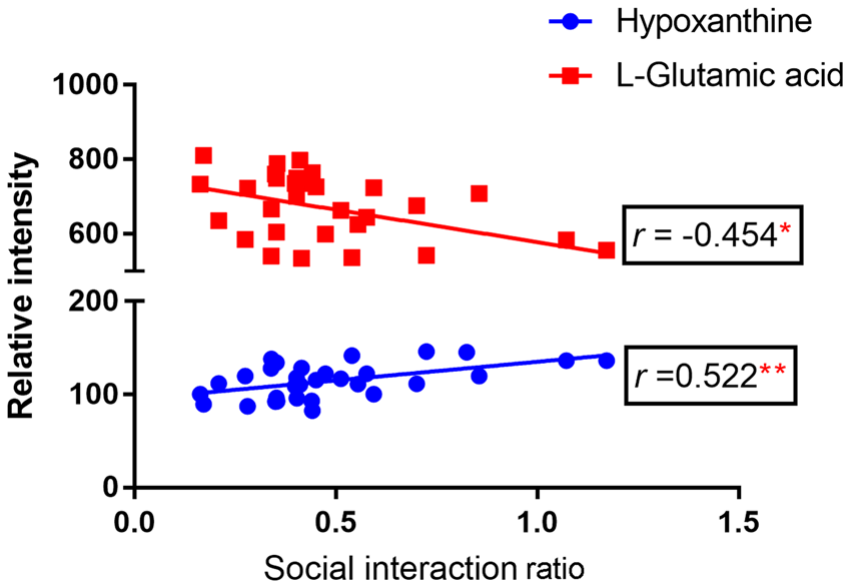
Correlation of differential metabolites with the social interaction ratio (*n* = 31). *P = 0.010 (L- glutamine vs. social interaction ratio); **P = 0.003 (hypoxathine vs. social interaction ratio), by Pearson’s test.

Pearson’s correlative analysis of all the differential metabolites (altered by DG and VLX) and the SI ratios revealed potential relationships between metabolites and SI ratios. Only L-glutamic acid and hypoxanthine showed a significant correlation with the SI ratio (Fig. 11). L-glutamic acid was negatively correlated with the SI ratio (*r = *−0.454, *P = *0.010), however hypoxanthine was positively correlated with the SI ratio (*r = *0.522, *P = *0.003).

## Discussion

Depression is a complex and heterogeneous disorder and a major contributor to the global burden of disease, and our group has focused on this disease for many years^2, 5, 35–37^. The majority of recent studies on antidepressants have focused on tricyclic antidepressants and selective serotonin reuptake inhibitors. However, most of these drugs are characterized by low rates of response and severe adverse reactions. A recent study by our group showed that DG has antidepressant-like effects on baseline behaviours in the mouse^14^, suggesting that this alternative medicine might have therapeutic potential for treating depression.

In the present study, using a validated model of CSDS^32, 38^, we demonstrate, for the first time, that DG ameliorates behavioural deficits caused by chronic exposure to CSDS. Both DG and VLX attenuated CSDS-induced behavioural deficits, including the changes in sociability and vertical exploration behavior. Notably, DG also diminished despair behaviour.

Following CSDS, 77.5% of the defeated mice showed apparent social avoidance, characteristic of susceptible or depressed individuals. Susceptible individuals have been proved to display typical behavioural deficits, including social avoidance, anxiety-like behaviour, despair behaviour and anhedonia^33, 38^. In this study, only the susceptible mice were used to screen for antidepressant drugs in further experiments. Even after six weeks, the susceptible mice (DEP group) still showed social deficits (SI ratio < 1; Fig. 3A), indicating high validity of the CSDS model. VLX is a clinically common antidepressant, but there is only a few definitive evidences that it attenuates CSDS-induced social deficits^17^. Herein, we both tested the antidepressant effects of DG and VLX in the susceptible mice after CSDS.

Our previous study revealed that 2 weeks of DG and VLX treatment led to antidepressant-like effects in mice^16^. However, in the present study, we found no differences in social behaviour (typical depressive behaviour associated with the CSDS model) among the three groups after 2 weeks of treatment. In view of the administration methods described by two independent studies were entirely consistent. The reason why the drugs have no antidepressant effect may be that this study is based on the CSDS model. Similarly, Venzala *et al*. (2012) also demonstrated that venlafaxine showed antidepressant-like activity in the depressed mice after 30 days of treatment^17^. Therefore, model differences should be taken into account when attempting to replicate such results. After 6 weeks of chronic treatment with DG or VLX, the social deficits were significantly attenuated. It suggested that DG and VLX began to play a significant antidepressant effect after 6 weeks. In this case, we also assessed other depression-like behaviours. In the SPT, DG and VLX did not affect the hedonic response. In the FST and TST, DG decreased the immobility time in the FST, indicating that it impacts partial despair behaviour. In addition, DG and VLX induced more frequent rearings in the OFT, indicating a greater exploratory behaviour. However, neither DG nor VLX had an anxiolytic effect in the OFT or EPM. Furthermore, neither of these drugs influenced memory function in susceptible mice in the Y-maze test.

The PFC is an important brain centre that controls stress responses and regulates susceptibility and resilience after CSDS^26, 39, 40^. We used the high-resolution and reliable GC-MS-based metabolomics approach to study the mechanisms of action of DG and VLX in the PFC of mice. We identified 19 and 30 differential metabolites that definitively discriminated mice in the DG-treated and VLX-treated groups, respectively, including 15 common differential metabolites showing the same trend (Fig. 6). Despite the commonalities, the two groups were clearly distinguishable (Fig. 5C,F), with 17 differential metabolites between them (Supplementary Table S1). This finding suggests that while both DG and VLX could attenuate stress-induced behavioural deficits, their molecular mechanisms of action differ substantially.

To provide further insight into the underlying antidepressive mechanisms, the significantly differential metabolites were analyzed using the IPA knowledgebase to identify their biochemical functions and pathways. This analysis revealed that “tRNA charging” was the top canonical pathway for both DG and VLX. TRNA charging plays an important role in the expression of genes, consistent with the “protein biosynthesis” result obtained with MetaboAnalyst (Fig. 9A,B). This suggests that both DG and VLX affect the biosynthesis of proteins. Interestingly, among all metabolites (L-glutamic acid, L-tyrosine, L-phenylalanine, L-alanine, L-proline, L-threonine, L-asparagine, L-isoleucine, L-histidine, L-lysine, L-aspartic acid, L-arginine, L-cysteine, L-glutamine, L-leucine, L-methionine, L-valine, L-tryptophan, D-serine) involved in “protein biosynthesis”, only L-glutamic acid was downregulated in both the DG and VLX groups; the remaining metabolites showed upregulation or no change. Based on these results, we speculate that DG and VLX exert their antidepressive effects, at least in part, by enhancing protein biosynthesis. Another important canonical pathway was “purine nucleotides degradation”, which includes phosphate, adenosine 5ʹ-monophosphate, inosinic acid, inosine and hypoxanthine (Fig. 10). This pathway and pyridine metabolism (Fig. 9D) are both nucleotide metabolism pathways. From the schematic diagram (Fig. 10), we can see that upstream metabolites were downregulated, while downstream metabolites were upregulated, indicating that DG and VLX enhance nucleotide degradation. Nucleotide degradation is an importance source of raw materials for the synthesis of nucleic acids and for enhancing pentose phosphate metabolism. Protein biosynthesis and nucleotide metabolism are the two most important requirements for the proliferation of cells, and thus likely accounting for the enhanced cell proliferation in this study. Furthermore, compared with VLX, the impact of DG on “growth of organism” (Supplemental Figure S4) and “protein biosynthesis” (Supplemental Figure S9) was weaker. Among the differential metabolites involved in these functions, hypoxanthine, which belongs to nucleotide metabolism, showed a significant positive correlation with the SI ratio (Fig. 10). It is worth noting that the upregulation of hypoxanthine in both the DG and VLX groups is predicted to enhance the proliferation of cells, as shown by IPA in Fig. 8.

Another three canonical pathways—“superpathway of citrulline metabolism”, “asparagine biosynthesis I” and “l-glutamine biosynthesis II” —“which belong to “alanine, aspartate and glutamate metabolism” were also influenced by DG or VLX treatment. The schematic diagram shows that aspartate metabolism and glutamate metabolism were significantly affected pathways, which include 9 differential metabolites. These two pathways are associated with neurotransmitter metabolism. L-glutamic acid, a significant excitatory neurotransmitter in the mammalian CNS, was decreased both in DG and VLX- treated mice. Previous studies by others and our research group have revealed that glutamate metabolism played a significant role in depression^33, 39, 41, 42^. Lower L-glutamic acid levels are detected in the PFC of depression-resistant mice^33^, and the glutamatergic system has been effectively used as a target for the treatment of depression^43, 44^. In our study, the changes in glutamate metabolism indicate that DG and VLX might exert their antidepressive effects by reducing glutamate excitotoxicity. Aspartate metabolism also helped discriminate the metabolic profiles of DG and VLX-treated mice from that of controls. The biosynthesis of asparagine is essential for the development and function of the brain^45^. Previous studies have suggested that asparagine levels may predict the response to antidepressant treatment^19, 46^. In addition, the “superpathway of citrulline metabolism”, including (including adenosine 5ʹ-monophosphate, L-aspartic acid, L-glutamic acid, L-glutamine, L-proline, phosphate and fumaric acid) is also mainly involved in aspartate and glutamate metabolism. L-asparagine is biosynthesized from L-aspartic acid by asparagine synthetase using L-glutamine as an amino group donor. The results are in line with the assumption that the decline in glutamate metabolism strengthens aspartate metabolism. Additionally, among the differential metabolites, L-glutamic acid showed a significant negative correlation with the SI ratio (Fig. 10). This suggests that the downregulation of L-glutamic acid in both the DG and VLX groups might play an important role in mediating the antidepressive effects of these drugs.

The pathway “tyrosine degradation I” was significantly affected in DG-treated mice, but not in VLX-treated mice. The upregulation of L-tyrosine might enhance its degradation. L-tyrosine is the precursor of dopamine. Consistent with this finding, our lab’s previous study showed that fluoxetine upregulates tyrosine in astrocytes^19^, and that tyrosine is downregulated in the brain of a rat model of depression^41^. Therefore, the observed tyrosine upregulation in DG-treated mice suggests that DG therapy might attenuate despair behaviours through a tyrosine-related mechanism.

The initial IPA results showed that “amino acid metabolism, small molecule biochemistry, cell-to-cell signaling and interaction” (Supplemental Figure S8) play key roles in discriminating the two antidepressants. However, both amino acid metabolism and small molecule biochemistry were influenced by DG and VLX. Therefore cell-to-cell signalling and interaction are critical for discriminating the two antidepressants. The results from the IPA database analysis predicted that ERK1/2, akt, Mapk, EGFR and NTS,which have been reported as highly correlated with the pathogenesis of depression, are hub genes that discriminate the effects of DG and VLX on the PFC in mice (Supplemental Figure S8).

Finally, to better clarify the antidepressant effect of DG, we compared the significant metabolites in this study with those documented in our previous report (Supplemental Figure S10)^14^. Since the models and brain regions used were different, there were only three common metabolites (pyroglutamic acid, asparagine, and inosine). Pyroglutamic acid is a downstream metabolite of glutathione, which serves as an antioxidant. The downregulation of pyroglutamic acid reflects a lower degradation of glutathione. Evidence has shown that depressed patients and animals subjected to stress have an excessive oxidation status^47, 48^. Therefore, DG may induce an antidepressant effect through its antioxidant properties. Asparagine is a neurotransmitter-related amino acid^14^. The inconsistency regarding asparagine changes in the two studies may be due to differences in asparagine levels in distinct brain regions. Accumulating evidence has suggested that inosine has promising antidepressant effects in animal models of depression^49–51^. Muto *et al*. revealed that inosine may produce antidepressant-like effects through neuroprotection in CSDS mice^49^, and Kaster *et al*. also reported that inosine may possess antidepressant-like effects in the FST and TST in mice through the activation of adenosine receptors^51^.Consequently, it is an interesting finding that DG could display antidepressant effects through upregulating inosine level.

There are several limitations of our study that should be acknowledged. First, the number of samples in this study was relatively small. A study with a larger sample size and using a targeted metabolomics approach is required for better assessing the effects of DG and VLX on depression-like behaviours. Second, the comparisons in this study were based on depressed mice. An additional comparison between naive and depressed mice might help clarify the antidepressive mechanisms of action of DG and VLX. Finally, administering ginkgolides A, B, C and K individually might help distinguish the antidepressive effects of these component ginkgolides.

## Conclusion

In conclusion, both DG and VLX effectively attenuate stress-induced social deficits, despair behaviour and exploratory behaviour. in mice. Comparative metabolomic profiling of the PFC revealed that DG and VLX have both common and distinct antidepressive molecular mechanisms of action. Both antidepressants influence protein biosynthesis and nucleotide metabolism to enhance cell proliferation, but DG has a weaker impact on these processes than VLX. Glutamate and aspartate metabolism might play an important role in the antidepressive effects of DG and VLX. Tyrosine degradation and cell-to-cell signalling and interaction play key roles in discriminating the two antidepressants. Moreover, interestingly, L-glutamic acid was negatively correlated with the SI ratio, while hypoxanthine was positively correlated with the SI ratio. Investigating the metabolic changes produced by DG and VLX in the PFC should provide important insight into the cell and molecular changes underlying the therapeutic actions of these medicines, as well as help advance antidepressant drug discovery efforts.

## Materials and Methods

### Animals and ethics statement

Male C57BL/6 mice (aged approximately 6–8 weeks and weighing 20–25 g) and male CD-1 (ICR) mice (aged approximately 18–20 weeks and weighing 35–40 g) were obtained from the laboratory animal centre of Chongqing Medical University (Chongqing, China). Mice were housed individually in standard polypropylene cages with wood shavings, under a 12-h dark/light cycle (lights on at 8:00 am) and a temperature (23 ± 2 °C) and relative humidity controlled environment. Mice had unlimited access to food and water. All animal experimentation procedures were performed according to the recommendations of the Guide for the Care and Use of Laboratory Animals, and were approved by the Ethics Committee of Chongqing Medical University (Permit number: 20120126).

### CSDS and drug treatments

The CSDS procedure was performed as previously reported^26, 32, 33^. During the 10-day defeat period, the C57BL/6 mouse was introduced to a novel aggressive resident CD-1 mouse for 10 min every day. Immediately after the social defeat session, the resident CD-1 mouse and the C57BL/6 intrude mouse were housed in one half of the cage which were separated by a perforated transparent Plexiglas divider for continued sensory contact. Then, 24 h after the last stress session, the C57BL/6 mouse was housed individually, and the SI test was performed to identify subgroups of mice that were susceptible or resilient to CSDS. This was accomplished by placing mice in an open field box (44 cm × 44 cm × 30 cm) with an empty perforated plastic box (10 cm × 7 cm × 18 cm) located at one end. The movement of the mice was videotaped for 2.5 min, followed by 2.5 min in the presence of an unfamiliar CD-1 mouse confined in the perforated plastic box. The movements were quantified by an animal movement analysis system (SMART, Panlab SL, Barcelona, Spain). The time spent in the interaction zone (defined as the 8-cm-wide area surrounding the perforated plastic box) was recorded. The SI ratio = time spent in an interaction zone with a CD-1 mouse/time spent in an interaction zone without a CD-1 mouse. An SI ratio of 1 was set as the cut-off. Mice with scores <1 were defined as susceptible to social defeat stress and those with scores >1 were defined as resilient. Only susceptible mice were used in the subsequent experiments.

After the CSDS procedure, the susceptible mice were randomly divided into the following three groups: (1) depressed group (DEP; 0.9% NaCl solution; *n* = 10), (2) DG group (12.18 mg/kg; Diterpene Ginkgolides Meglumine Injection, provided by Jiangsu Kanion Pharmaceutical Co., Ltd, diluted in 0.9% NaCl solution; *n* = 11) and (3) VLX group (16 mg/kg VLX, diluted in 0.9% NaCl solution, *n* = 11). Mice were given intraperitoneal injections daily in a volume of 200 μl. The doses were based on our previous study^14^.

### Behavioural testing

After drug treatment, a series of behavioural studies were performed during the daytime (lights-on period) under conditions of dim light and low noise. All behavioural tests were videotaped and quantified by an animal movement analysis system (SMART, Panlab SL, Barcelona, Spain). The behavioural tests used are well established^2, 4, 14, 52–54^. The experimental timeline and study design are shown in Fig. 1.

*SPT:* The SPT was performed to evaluate the anhedonic response after treatment. After 24 h deprivation of water and food, the two-bottle preference test was conducted which all mice had access to water and 1% sucrose water for 24 h. Sucrose preference = sucrose intake/(sucrose intake + water intake) × 100%.

*OFT:* Mice were placed individually in the centre of an open field box (44 cm × 44 cm × 30 cm) and allowed 5.5 min to move freely. After 0.5 min of adaptation, the distance travelled and the duration spent in the centre were analyzed during a period of 5 min using the SMART system. Rearings were scored in a blind manner by an experimenter. After each test, the field was cleaned with a 75% ethanol solution.

*EPM:* The EPM consisted of 4 arms (30 cm long and 6 cm wide), with two open arms (no side or end walls) and two closed arms (15-cm-high side walls and 15-cm-high end walls). A central 6 cm × 6 cm square platform allowed access to all arms. Mice were placed in the central square, and after 0.5 min of adaptation, the distance travelled, the number of entries and the duration in each arm during a 5-min period were analyzed using the SMART system. The degree of anxiety was assessed by calculating the time or percentage of time spent in the open arms.

*TST:* Mice were individually hung on a hook by their tails using a small piece of adhesive tape (2 cm from the tip of the tail). Every test session lasted 6 min, and the last 5 min were scored by SMART to assess immobility. Animals were considered to be immobile only when they remained hung on the hooks passively and were completely motionless.

*Y-maze:* The Y-maze apparatus consisted of three arms intersecting at 120° (45 cm long, 10 cm wide and 29 cm high). Mice were placed at the end of one arm and allowed to freely explore the three arms for 8 min. The sequence and the total number of arms entered was noted. Percentage of spontaneous alternation was defined as [(number of alternations)/(total number of arm entries − 2)] × 100%.

*FST:* The mice were placed individually in a clear Plexiglas cylinder (30 cm in height, 15 cm in diameter) filled with water (25 ± 1 °C) to a height of 15 cm. Test sessions lasted for 6 min, and the last 5 min were scored by SMART to assess immobility. Animals were considered to be immobile only when there were only occasional slight movements (required to keep the head out of the water).

### GC-MS metabolomics analysis

The procedure for GC-MS metabolomics was conducted in accordance with our previous study^55^. The day after the completion of the behavioral tests, the mice were anesthetized with chloral hydrate and decapitated, and the whole brains were removed. The PFC was separated from the brain, weighed, rapidly frozen with liquid nitrogen, and stored at −80 °C until analysis.

Prior to GC–MS metabolomics analysis, each 20 mg piece of PFC was ground with 400 μL of a methanol and water (4:1) solution containing L-2-chlorophenylalanine (0.075 mg/mL, internal standard) using a tissue homogenizer. The mixture was sonicated and centrifuged, and the liquid supernatant was gathered and lyophilized. Then, the dried extract was derivatized into its methoxime derivatives by a reaction with methoxamine hydrochloride in pyridine (15 mg/ml). Subsequently, the solution was derivatized with BSTFA (1% TMCS) and *n*-hexane at 70 °C for 60 min, and placed at room temperature for 30 min before analysis.

Metabolic profiling of the PFC in a discovery set based on non-targeted analysis was performed using the Agilent GC system 7890 A equipped with a 5975 C mass-selective detector system. Typical GC settings and MS parameters were used. Briefly, the temperatures of the injector, the EI iron source and the quadrupole rods were set at 280 °C, 230 °C and 150 °C, respectively. High-purity helium carrier gas flowed at a steady rate of 6 mL/min. A 0.5-µL aliquot of each sample was used for metabolite separation. Column temperature was initially kept at 70 °C for 2 min, increased to 160 °C at a rate of 6 °C/min, then raised to 240 °C at a rate of 10 °C/min, and finally raised to 300 °C at a rate of 20 °C/min. This temperature was maintained for 6 min. MS spectra were acquired from m/z 50 to 600.

The chromatographic peaks in the total ion current (TIC) chromatograms represent corresponding metabolites, and their relative concentrations can be detected using the peak area normalization method. In the present study, the TIC from the three group PFC samples revealed strong signals for analysis, large peak capacity and good retention time reproducibility (Supplemental Figure S1). 646 individual peaks were detected in each group. These peaks were annotated by comparing the accurate mass (m/z) and retention time (RT) from the National Institute of Standards and Technology online databases. The relative intensities of these metabolites were used in the subsequent multivariate statistical analysis.

### Statistical analysis

All data from the behavioural tests were expressed as means ± SEM. The time in the interaction zone (SI test) data were analysed using two-tailed *t*-tests for comparisons between target and no target, and other data were analyzed by one-way analysis of variance (ANOVA), followed by the post hoc least significant difference test or Dunnett’s test, depending on the homogeneity of variances, with repeated measures as indicated. Significance was denoted at a *P-*value* < *0.05. The SPSS 21.0 software package (IBM, Armonk, NY, USA) was used for all statistical tests. The statistical analysis of the correlation between significant metabolites and the SI score was performed using Pearson’s test. For plotting all data, Graphpad Prism 7.0 (GraphPad Software, Inc. USA) was used.

GC–MS-based metabolomics data were converted into a NetCdf file format using TagFinder^56^. The peak indexes (RT-m/z), sample names and normalized peak intensities were imported into SIMCA 14.1 (MKS Umetrics, AB). Multivariate statistical analyses, including principal component analysis (PCA) and pair-wise orthogonal projections to latent structures discriminant analyses (OPLS-DA) with Pareto scaling spectral data was performed to visually discriminate samples in the CON, DG and VLX groups. R^2^Y and Q^2^ were used to quantify the quality of the model. Significant metabolites were indicated by VIP > 1 (variable importance in the projection value) and a *P-*value* < *0.05 (obtained from two-tailed Student’s *t*-test). Cytoscape software 3.4.0 was used to build the correlation network between these differential metabolites and DG treatment or VLX treatment.

### Metabolic functions and pathways analysis

The module for metabolite set enrichment analysis (MSEA) and the module for pathway analysis in the MetaboAnalyst 3.0^57^ website was used to identify the metabolic pathways significantly affected by these metabolites. We used MetaboAnalyst to generate a heat map of all the different metabolites. IPA software (Qiagen, Redwood City, CA, USA) was used to analyze the different metabolites to obtain information about predicted molecular pathways and biological functions, as described in http://www.ingenuity.com. IPA Molecule Activity Predictor was used to predict the activity of molecules highly connected to these networks. The z-score was used to identify significant biological functions and pathways, and a z-score >2 or <−2 indicated significance. Moreover, the network score was used to determine the relevance between metabolites and networks.

## Electronic supplementary material

Supplementary Information
