# Supplementary material for: Insight into the metabolic mechanism of Diterpene Ginkgolides on antidepressant effects for attenuating behavioural deficits compared with venlafaxine
Source: Sci Rep. 2017 Aug 29;7:9591. doi: 10.1038/s41598-017-10391-1 (PMC5575021; doi:10.1038/s41598-017-10391-1)
Supplement: Supplementary file 1 — Supplementary Information [file 41598_2017_10391_MOESM1_ESM.doc]

### Insight into the metabolic mechanism of Diterpene Ginkgolides on antidepressant effects for attenuating behavioural deficits compared with venlafaxine

Shunjie Bai^1,2,3,4*^, Xiaodong Zhang ^2,3,6*^, Zhi Chen ^1,2,3,4*^, Wei Wang ^1,2,3*^, Qingchuan Hu ^2,3,4*^, Zihong Liang ^2,3,5^, Peng Shen ^2,3,6^, Siwen Gui^2,3^, Li Zeng^2,3,6^, Zhao Liu^2,3,6^, Jianjun Chen^2,3^, Xiongfei Xie ^2,7^, Hua Huang ^2,3,8^, Yu Han ^2,3,6^, Haiyang Wang ^2,3^, and Peng Xie ^1,2,3,4,6**^

^1^Department of Neurology, Yongchuan Hospital, Chongqing Medical University, Chongqing, China

^2^Chongqing Key Laboratory of Neurobiology, Chongqing, China

^3^Institute of Neuroscience and the Collaborative Innovation Center for Brain Science, Chongqing Medical University, Chongqing, China

^4^Key Laboratory of Laboratory Medical Diagnostics of Education, Department of Laboratory Medicine, Chongqing Medical University, Chongqing, China

^5^Department of Neurology, The Inner Mongolia Autonomous Region people's Hospital, Hohhot, Inner Mongolia, China

^6^Department of Neurology, The First Affiliated Hospital of Chongqing Medical University, Chongqing, China

^7^Department of Radiology, The First Affiliated Hospital of Chongqing Medical University, Chongqing, China

^8^Department of Neurology, The Second Affiliated Hospital of Chongqing Medical University, Chongqing, China

^*^These authors contributed equally to the manuscript.

^**^**Corresponding author:** Professor Peng Xie, Department of Neurology, Yongchuan Hospital, Chongqing Medical University, Chongqing 402460, China

E-mail: [xiepeng@cqmu.edu.cn](mailto:xiepeng@cqmu.edu.cn) Tel.: +86-23-68485490; Fax: +86-23-68485111.

Table S1. Key differential metabolites in the comparison of DG and DEP groups in mice PFC

| **Metabolites** | **r.t(min)** | **Mass** | **Fold change** | **VIP score** | **t-test (*p*)** |
| --- | --- | --- | --- | --- | --- |
| Inosinic acid | 33.36 | 315 | 0.43 | 2.69 | 0.006 |
| Oxamic acid | 8.52 | 192 | 0.50 | 4.26 | 0.007 |
| Adenosine 5'-monophosphate | 34.04 | 315 | 0.69 | 1.88 | 0.021 |
| Glucose-1-phosphate | 18.82 | 217 | 0.79 | 1.13 | 0.008 |
| Cysteamine | 6.83 | 188 | 0.86 | 4.14 | 0.044 |
| Fumaric acid | 6.65 | 245 | 0.87 | 1.02 | 0.013 |
| L-Glutamic acid | 15.77 | 246 | 0.89 | 11.31 | 0.000 |
| Pyroglutamic acid | 13.73 | 156 | 0.95 | 9.84 | 0.033 |
| L-Phenylalanine | 15.90 | 218 | 1.08 | 1.37 | 0.004 |
| L-Tyrosine | 22.26 | 218 | 1.09 | 1.76 | 0.019 |
| Arachidonic acid | 28.72 | 106 | 1.09 | 1.12 | 0.019 |
| Isoleucine | 9.23 | 158 | 1.09 | 1.41 | 0.020 |
| L-Valine | 7.96 | 144 | 1.10 | 2.04 | 0.005 |
| L-Proline | 9.36 | 142 | 1.13 | 2.46 | 0.029 |
| Hypoxanthine | 19.61 | 265 | 1.19 | 5.30 | 0.000 |
| Uracil | 10.02 | 241 | 1.19 | 1.65 | 0.000 |
| Inosine | 30.44 | 259 | 1.25 | 4.55 | 0.001 |
| Beta-Alanine | 6.67 | 102 | 1.34 | 5.10 | 0.028 |
| L-Asparagine | 12.34 | 128 | 1.60 | 1.14 | 0.004 |

Table S2. Key differential metabolites in the comparison of VLX and DEP groups in mice PFC

| **Metabolites** | **r.t(min)** | **Mass** | **Fold change** | **VIP score** | **t-test (*p*)** |
| --- | --- | --- | --- | --- | --- |
| Inosinic acid | 33.36 | 315 | 0.10 | 2.36 | 0.000 |
| L-2,4-diaminobutyric acid | 30.15 | 301 | 0.40 | 1.05 | 0.000 |
| Adenosine 5'-monophosphate | 34.04 | 315 | 0.47 | 1.87 | 0.000 |
| O-Phosphoethanolamine | 19.11 | 315 | 0.56 | 1.33 | 0.036 |
| Oxamic acid | 8.52 | 192 | 0.61 | 2.09 | 0.030 |
| Cytosine | 27.29 | 131 | 0.65 | 3.30 | 0.012 |
| Dihydrouracil | 17.07 | 243 | 0.67 | 1.05 | 0.001 |
| Phosphate | 14.81 | 315 | 0.68 | 1.65 | 0.008 |
| Lipoamide | 32.15 | 131 | 0.71 | 1.83 | 0.033 |
| L-Glutamic acid | 15.77 | 246 | 0.74 | 11.65 | 0.000 |
| Pyroglutamic acid | 13.73 | 156 | 0.90 | 10.89 | 0.000 |
| N-Acetyl-L-aspartic acid | 16.59 | 202 | 0.95 | 2.47 | 0.006 |
| Alanine | 6.44 | 116 | 1.13 | 3.81 | 0.002 |
| L-Tyrosine | 22.26 | 218 | 1.15 | 1.59 | 0.001 |
| Arachidonic acid | 28.72 | 106 | 1.16 | 1.04 | 0.001 |
| L-Phenylalanine | 15.90 | 218 | 1.16 | 1.27 | 0.000 |
| Methyl Phosphate | 7.38 | 241 | 1.17 | 2.59 | 0.010 |
| L-Aspartic acid | 13.67 | 232 | 1.19 | 9.41 | 0.005 |
| L-Valine | 7.96 | 144 | 1.21 | 2.02 | 0.000 |
| Isoleucine | 9.23 | 158 | 1.22 | 1.59 | 0.000 |
| L-Proline | 9.36 | 142 | 1.29 | 2.59 | 0.001 |
| D-Ribose | 16.91 | 103 | 1.33 | 1.70 | 0.000 |
| Hypoxanthine | 19.61 | 265 | 1.40 | 5.25 | 0.000 |
| Inosine | 30.44 | 259 | 1.40 | 3.86 | 0.000 |
| Uracil | 10.02 | 241 | 1.47 | 1.61 | 0.000 |
| 2'-Deoxycytidine-5'-triphosphate | 12.83 | 160 | 1.50 | 1.73 | 0.016 |
| L-Glutamine | 12.67 | 155 | 1.61 | 2.32 | 0.034 |
| Beta-Alanine | 6.67 | 102 | 1.64 | 4.06 | 0.027 |
| Gamma-Aminobutyric acid | 7.05 | 142 | 2.46 | 5.11 | 0.029 |
| Fructose 6-phosphate | 27.97 | 315 | 2.86 | 1.29 | 0.010 |

Table S3. Key differential metabolites in the comparison of DG and VLX groups in mice PFC

| **Metabolites** | **r.t(min)** | **Mass** | **Fold change** | **VIP score** | **t-test (*p*)** |
| --- | --- | --- | --- | --- | --- |
| Methyl Phosphate | 7.38 | 241 | 0.78 | 3.73 | 0.000 |
| D-Ribose | 16.91 | 103 | 0.81 | 1.63 | 0.001 |
| Uracil | 10.02 | 241 | 0.81 | 1.25 | 0.002 |
| Hypoxanthine | 19.61 | 265 | 0.84 | 3.85 | 0.000 |
| L-Aspartic acid | 13.67 | 232 | 0.88 | 8.39 | 0.024 |
| Inosine | 30.44 | 259 | 0.89 | 2.18 | 0.008 |
| Isoleucine | 9.23 | 158 | 0.91 | 1.23 | 0.003 |
| Alanine | 6.44 | 116 | 0.91 | 3.29 | 0.015 |
| L-Valine | 7.96 | 144 | 0.92 | 1.46 | 0.004 |
| Pyroglutamic acid | 13.73 | 156 | 1.07 | 8.76 | 0.028 |
| L-Glutamic acid | 15.77 | 246 | 1.20 | 9.58 | 0.000 |
| N-Acetyl-L-aspartic acid | 16.74 | 184 | 1.22 | 1.09 | 0.025 |
| Adenosine | 30.86 | 259 | 1.23 | 1.31 | 0.047 |
| Phosphate | 14.81 | 315 | 1.42 | 1.98 | 0.010 |
| Lipoamide | 29.45 | 131 | 1.47 | 1.75 | 0.014 |
| Gamma-Aminobutyric acid | 13.89 | 145 | 3.10 | 1.25 | 0.047 |
| Inosinic acid | 33.36 | 315 | 4.01 | 1.35 | 0.019 |


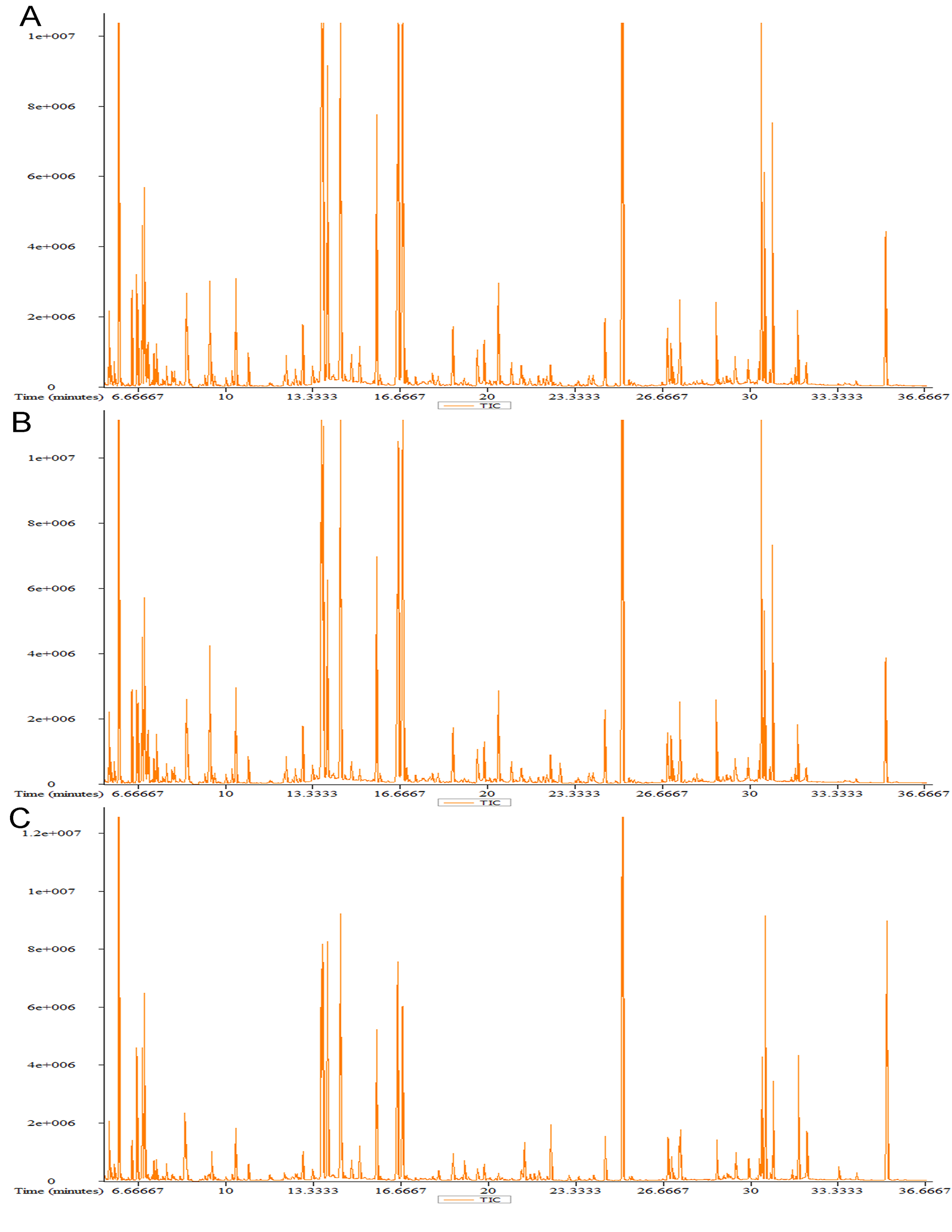


Figure S1 Representative GC–MS total ion chromatograms from a DG treated (A), a VLX treated (B) and a DEP (C) mice PFC.


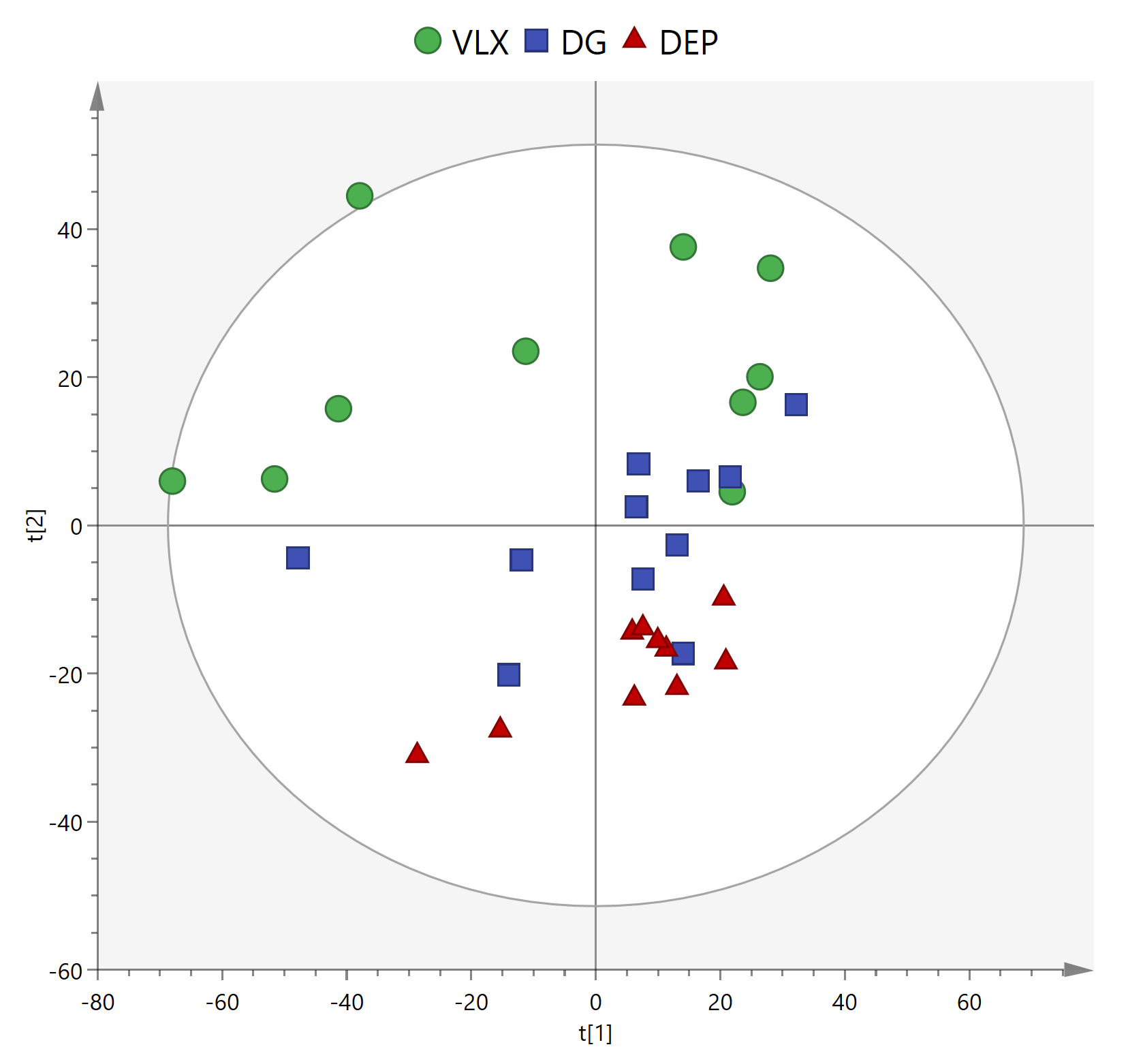


Figure S2 All analyzes groups PCA score plot of mice PFC samples. DEP, vehicle-treated mice; DG, diterpene ginkgolides-treated mice; VLX, venlafaxine-treated mice.


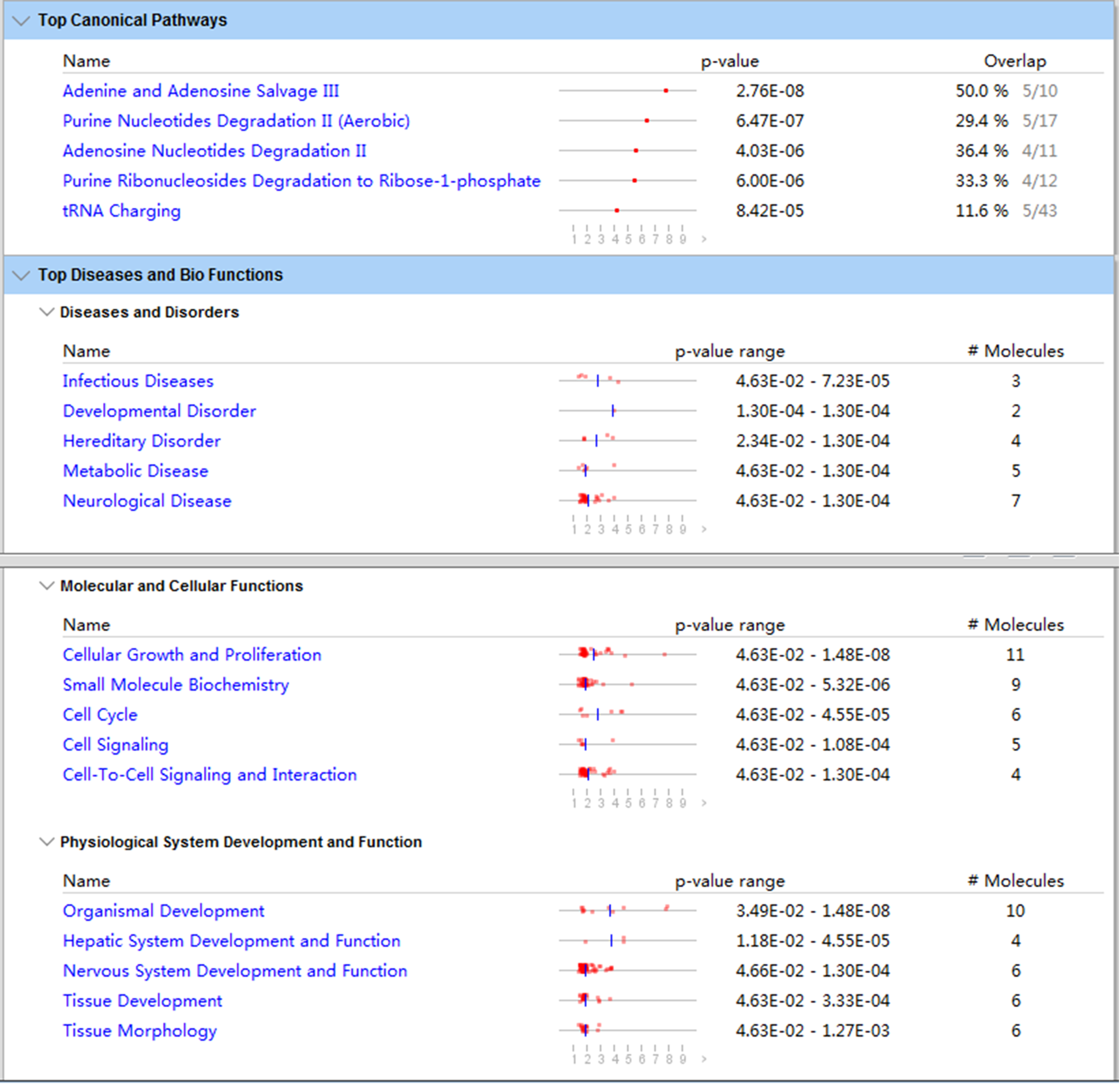


Figure S3 Top 5 “canonical pathways”, “diseases and disorders”, “molecular and cellular functions”, and “physiological system development and functions” enriched by observed metabolite alterations in the comparison of DG and VLX groups in mice PFC. DG, diterpene ginkgolides-treated mice; VLX, venlafaxine-treated mice.


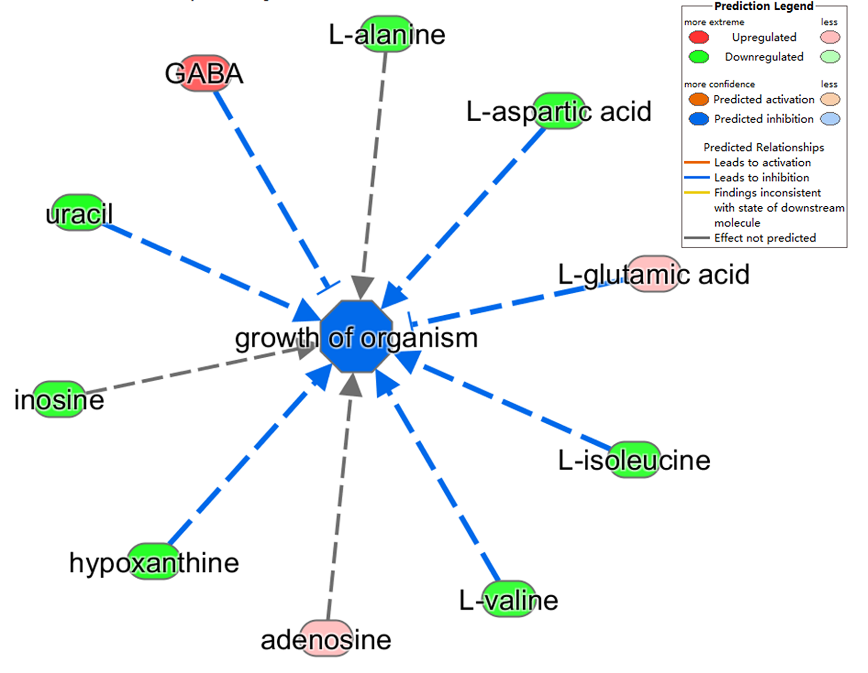


Figure S4 Predicted biological functions associated with the key differential metabolites in the comparison of DG and VLX groups in mice PFC.


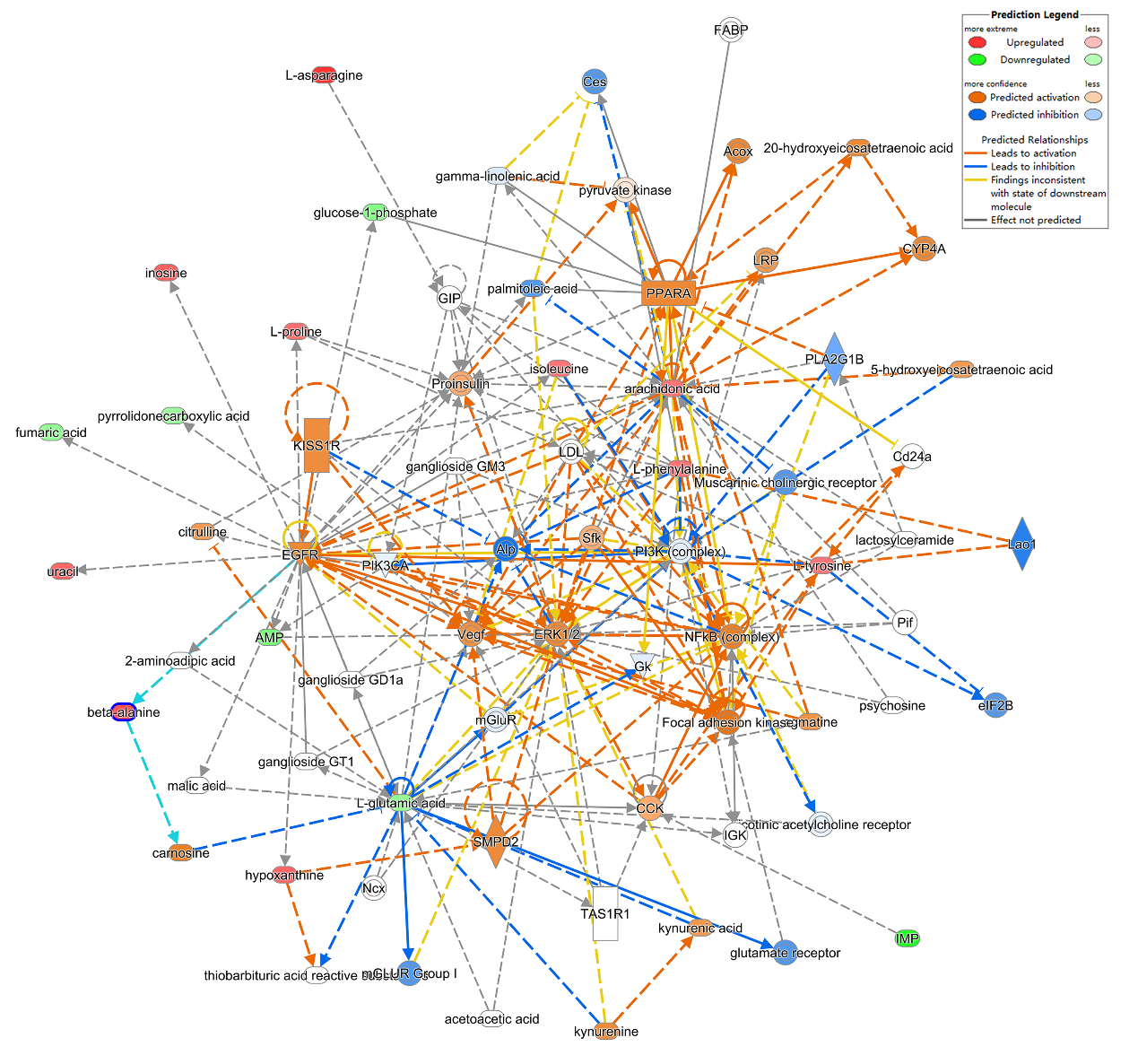


Figure S5 Network of “cellular compromise, lipid metabolism, small molecule biochemistry” associated with key differential metabolites in the comparison of DG and DEP groups in mice PFC with a score of 42 and 16 differential metabolites involved. DEP, vehicle-treated mice; DG, diterpene ginkgolides-treated mic.


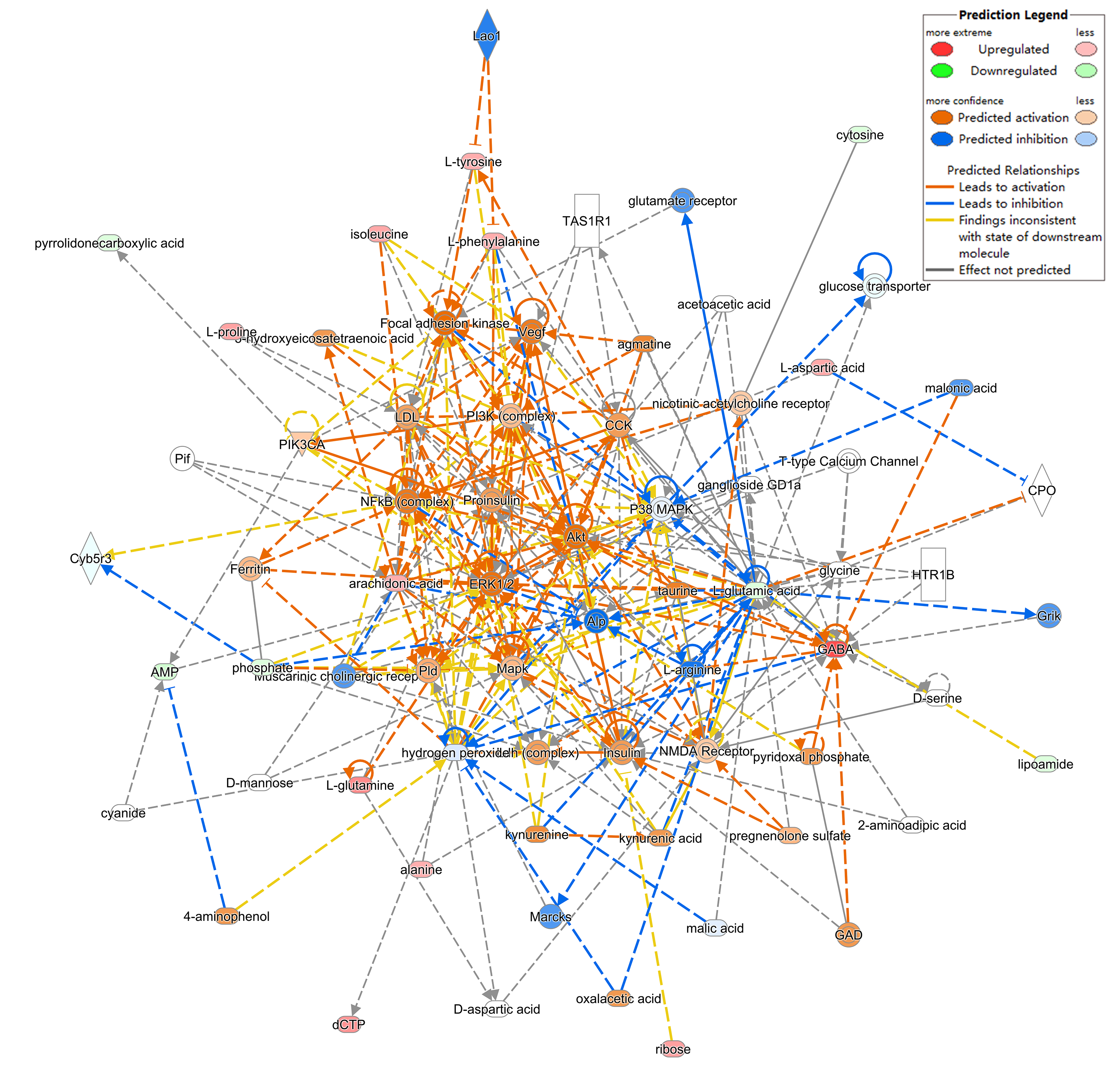


Figure S6 Network of “amino acid metabolism, small molecule biochemistry, cellular compromise” associated with key differential metabolites in the comparison of VLX and DEP groups in mice PFC with a score of 39 and 17 differential metabolites involved. DEP, vehicle-treated mice; VLX, venlafaxine-treated mice.


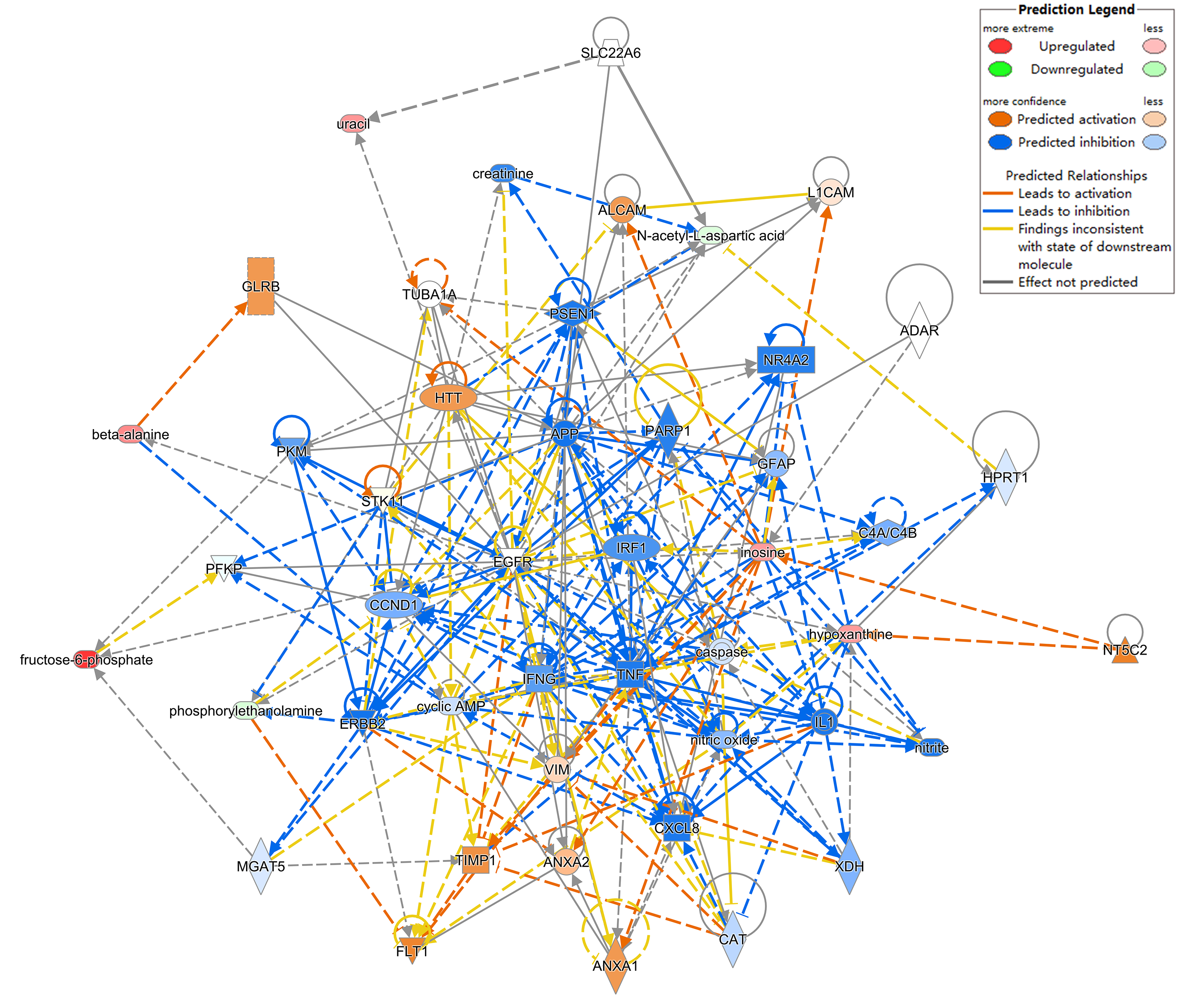


Figure S7 Network of “energy production, molecular transport, nucleic acid metabolism” associated with key differential metabolites in the comparison of VXL and DEP groups in mice PFC with a score of 14 and 7 differential metabolites involved. DEP, vehicle-treated mice; VLX, venlafaxine-treated mice.


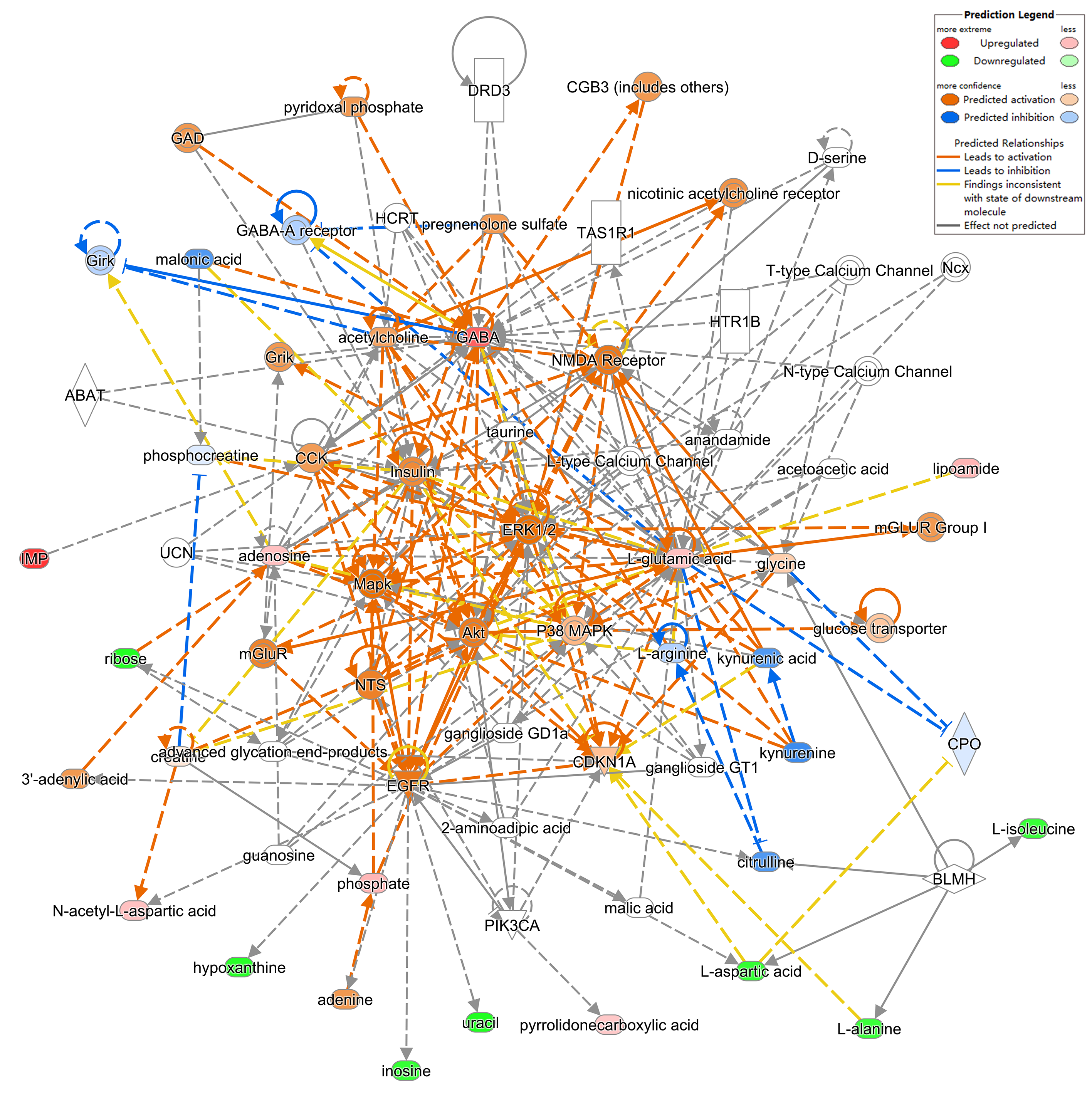


Figure S8 Network of “amino acid metabolism, small molecule biochemistry, cell-to-cell signaling and interaction” associated with key differential metabolites in the comparison of DG and VLX groups in mice PFC with a score of 39 and 15 differential metabolites involved. DG, diterpene ginkgolides-treated mice; VLX, venlafaxine-treated mice.


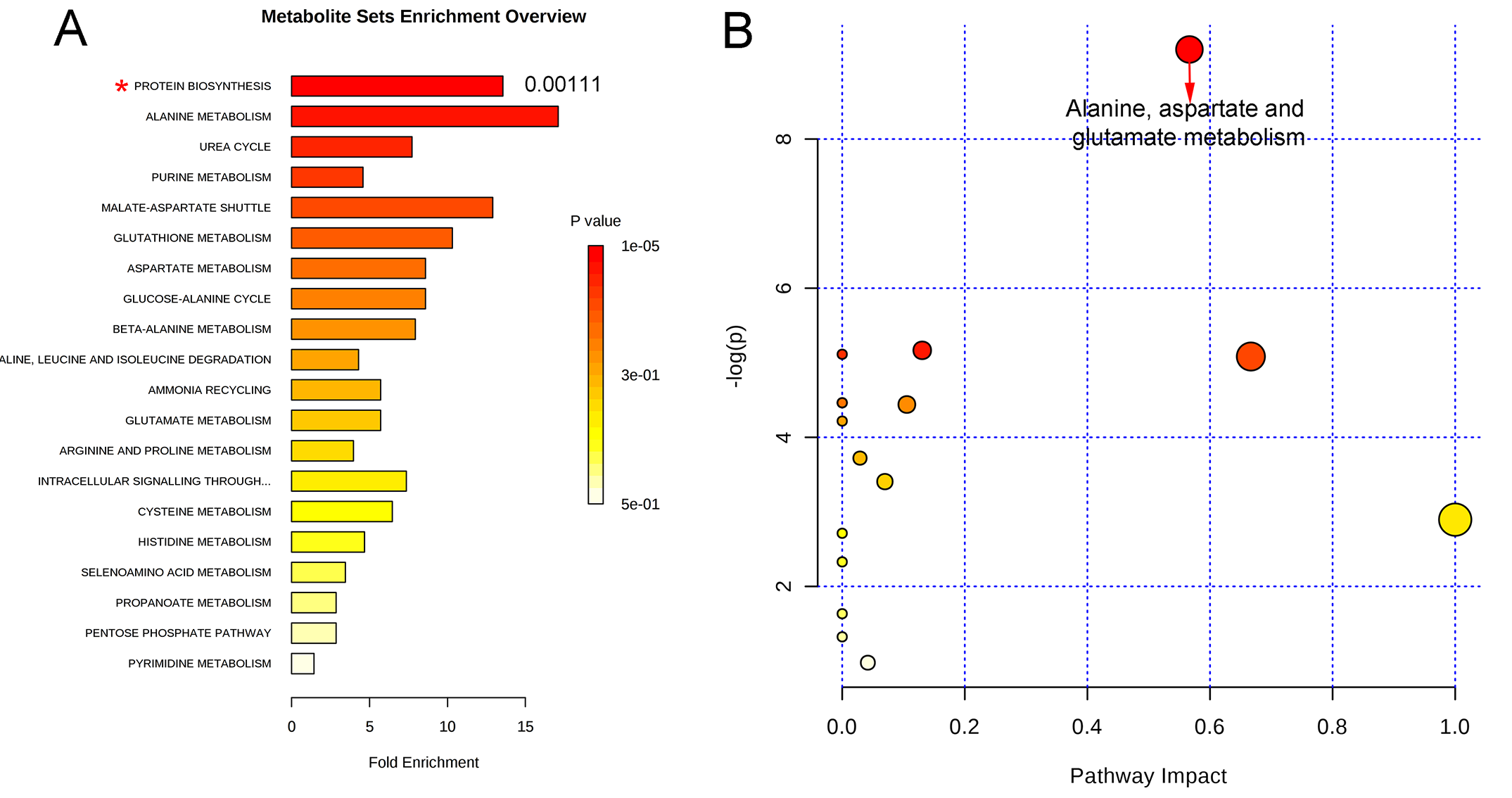


Figure S9 Significant biological functions (A) and Pathways (B) of Key differential metabolites in the comparison of DG and VLX groups in mice PFC. *: False discovery rate (FDR) < 0.05. →: False discovery rate (FDR) < 0.05 and impact value > 0. DG, diterpene ginkgolides-treated mice; VLX, venlafaxine-treated mice.


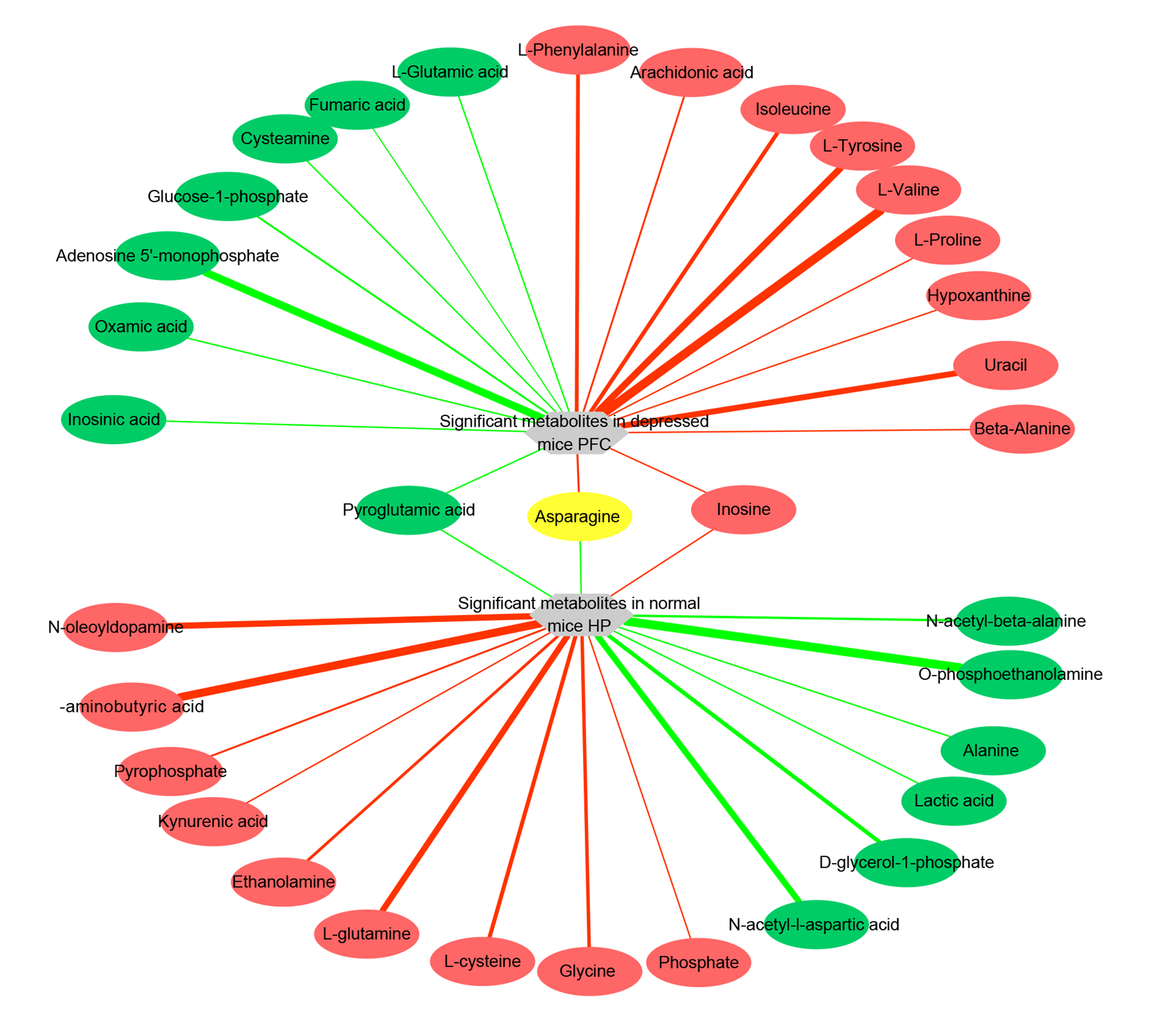


Figure S10 Correlation network of the significant metabolites in depressed mice PFC or normal mice HP. Red-coloured boxes and lines indicate upregulation in the diterpene ginkgolides treated group, while green-coloured boxes and lines indicate downregulation. The line width represents the VIP score of the differential metabolites. PFC, prefrontal cortex; HP, hippocampus.
